# Characterizing the Sulfated and Glucuronidated (Poly)phenol Metabolome for Dietary Biomarker Discovery

**DOI:** 10.1021/acs.jafc.4c12596

**Published:** 2025-03-03

**Authors:** Ioanna Tsiara, Belén Hervás Povo, Wafa Alotaibi, Paul Young Tie Yang, Ana Rodriguez-Mateos, Daniel Globisch

**Affiliations:** a Department of Chemistry - BMC, Science for Life Laboratory, Uppsala University, Box 576, Uppsala SE-75124, Sweden; b Department of Nutritional Sciences, School of Life Course and Population Sciences, Faculty of Life Sciences and Medicine, King’s College London, London WC2R 2LS, United Kingdom

**Keywords:** phase II modifications, metabolomics, gut microbiota, nutritional biomarkers, (poly)phenols

## Abstract

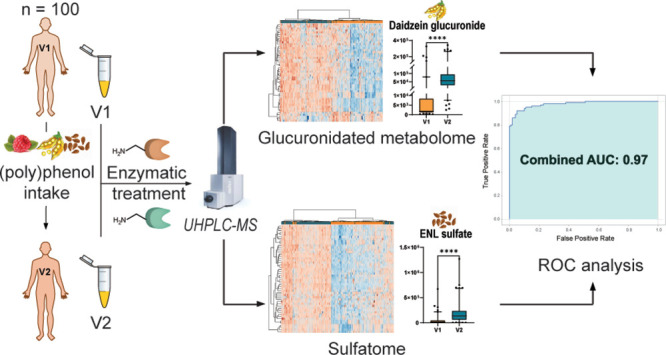

(Poly)phenols, bioactive compounds abundant in plant-based diets, have attracted interest for their potential role in preventing chronic diseases including cardiometabolic and neurodegenerative diseases. This study investigates the global sulfatome and glucuronidated metabolome in urine samples from 100 healthy adults collected pre- and postintervention following a 3-day (poly)phenol-rich intervention consisting of flaxseeds, raspberry powder, and soy milk. Using untargeted mass spectrometric metabolomics combined with selective phase II enzymatic treatment, we detected 156 sulfated and 143 glucuronidated metabolites in urine samples. Significant changes postintervention were observed for 91 sulfates and 94 glucuronides. Receiver operating characteristic curve analysis identified a combination of six polyphenol-derived key metabolites: glucuronidated daidzein and the sulfated compounds of pyrogallol, ferulic acid, 4-methoxyphenol, enterolactone, and resorcinol, which resulted in the best combination with the highest predictive AUC of 0.97. These findings underscore the utility of these metabolites as sensitive and selective biomarkers of (poly)phenol dietary intake.

## 1 Introduction

(Poly)phenols are a diverse group of naturally occurring compounds found abundantly in fruits, vegetables, tea, coffee, wine, and other plant-based foods that have gained increased attention due to their potential health benefits. These benefits are largely attributed to their cardioprotective, neuroprotective, and anti-inflammatory properties.^1,2^ Among the key metabolic processes that influence the bioavailability and biological activity of (poly)phenols in the human body are glucuronidation and sulfation. These metabolic clearance pathways are crucial for the detoxification and excretion of (poly)phenols.

(Poly)phenolic compounds can be described by the presence of multiple phenolic fused and/or connected rings in their structure. They are mainly divided into five categories: (i) flavonoids, which consist of three-membered ring structures with a central ring that can be oxidized or reduced, leading to different subclasses; (ii) phenolic acids, consisting of a benzene ring and a carboxylic acid; (iii) lignans, which consist of the dimerization of phenylpropanoids; (iv) stilbenes, which have two benzene ring structures connected by an alkene bond; and (v) tannins, complex phenolic compounds mainly found in vegetables, whose structure consists of polyhydroxylated flavonoid-analogue structures.^3^

**Figure 1:**
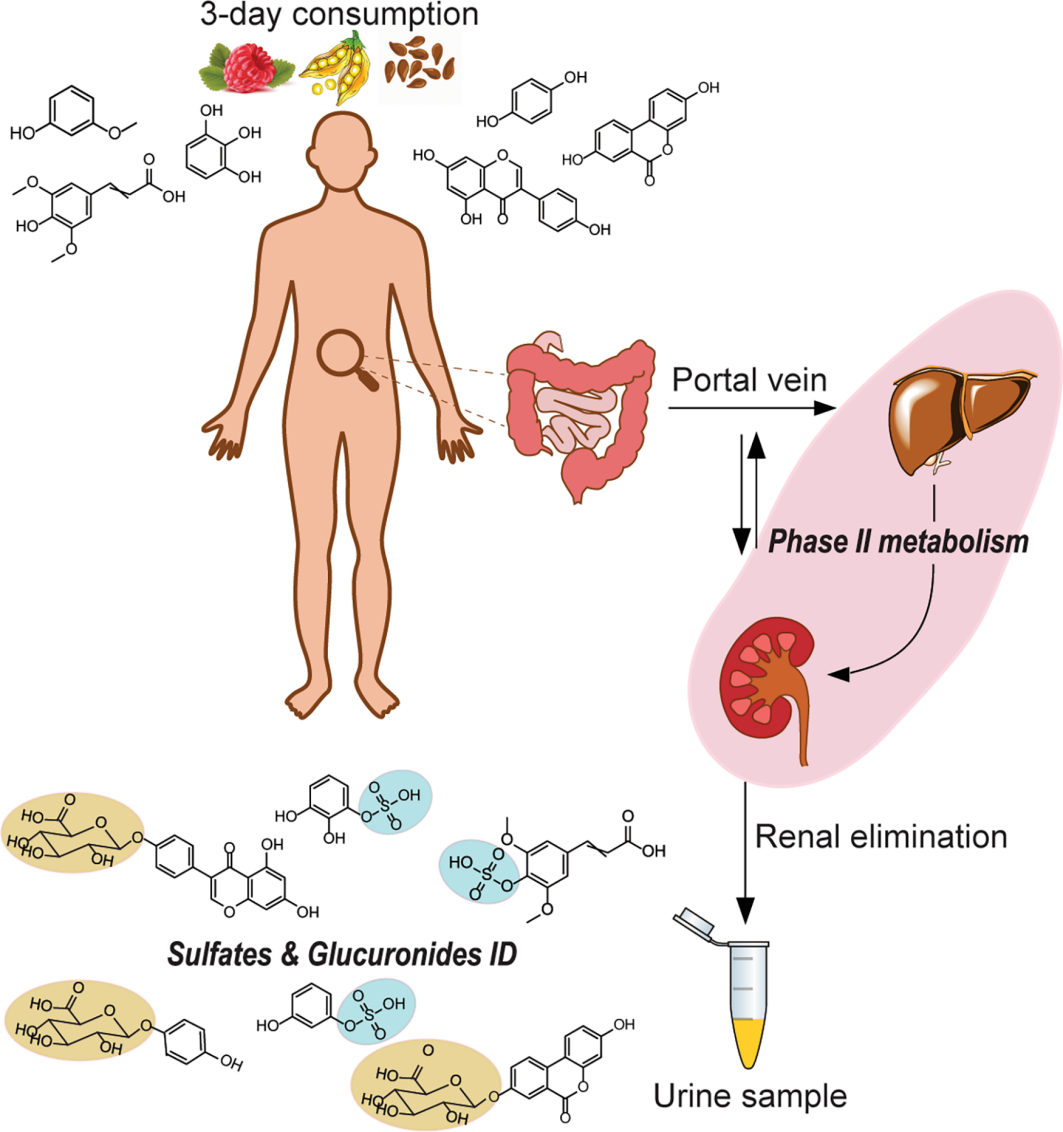
Biotransformation of dietary (poly)phenols in humans. Sulfates and glucuronides in urine samples are the end products of the conversion of dietary components in the liver and kidneys.

Dietary compounds are metabolized through human phase II modification processes to enhance their solubility and facilitate excretion from the human body. One major pathway is glucuronidation, primarily catalyzed by the enzyme family of uridine 5′-diphospho-glucuronosyltransferases (UGTs). UGTs mediate the conjugation of glucuronic acid to (poly)phenols, increasing their polarity for elimination via bile and urine (Figure 1). The second major phase II modification is sulfation, which involves the conjugation of the sulfate group to (poly)phenols catalyzed by sulfotransferases (SULTs). These processes not only aid the clearance of potentially toxic compounds but also modulate the biological activity of (poly)phenols, possibly enhancing their health-promoting effects. Urine samples are the main sample type for investigation of these compound classes as they can be found enriched and are the end point products of conversion and clearance.

In addition to these hepatic biotransformation processes, the gut microbiota play a pivotal role in the metabolism of dietary (poly)phenols through modification and degradation of metabolites. The complex community of microorganisms residing in the gastrointestinal tract can convert (poly)phenols through different biotransformation processes into metabolites of diverse scaffolds, which can alter their bioactivity profiles compared with the parent metabolite. Gut microbiota-mediated metabolism can enhance or impair the absorption of (poly)phenols changing their systemic availability and effects.^4^

The interplay among (poly)phenol consumption, glucuronidation, sulfation, and gut microbiota has not been elucidated in great detail yet due to their complex interactions. Previous studies have shown that individual differences in phase II metabolism can alter (poly)phenol bioavailability and biological activity.^5^ Furthermore, the gut microbiota composition influences (poly)phenol metabolism, as certain bacterial species can transform lignans, flavonoids, and ellagitannins into bioactive metabolites.^6^ Insights into this interaction are essential for optimizing the health benefits of (poly)phenol-rich diets. Emerging research suggests that the efficiency of glucuronidation and sulfation can vary significantly among individuals due to genetic, environmental, and dietary factors, thereby influencing the overall impact of (poly)phenols on health.^7^ Additionally, the composition and function of the gut microbiota can be influenced by the diet, lifestyle, and antibiotics, which also play a critical role in determining the metabolic fate of (poly)phenols.^8,9^

Understanding these complex metabolic interactions has been challenging due to the difficulties associated with the broad-scale analysis of glucuronidated and sulfated metabolites in mass spectrometry-based metabolomics. Until now, investigations of these metabolite classes have predominantly been conducted in a targeted manner for selected metabolite classes, as comprehensive analytical tools for both major phase II compound classes have been lacking. Various mass spectrometric methods have been utilized to investigate (poly)phenols; however, there is a strong need for more advanced and selective methodologies to investigate known and discover yet unknown phase II metabolites. To overcome these limitations, we have developed a series of enzymatic tools for the detailed investigation of a broad range of sulfated metabolites (sulfatome) in human samples.^10,11^ In this study, we have expanded these tools by utilizing a recombinant arylsulfatase and a new β-glucuronidase instead of crude enzyme mixtures, allowing for greater specificity. Additionally, we have explored the combined glucuronidation and sulfation profiles of a group of healthy individuals before and after the consumption of a (poly)phenol diet, providing a more comprehensive investigation of phase II metabolism.

## 2 Materials and Methods

### 2.1 Study Design

In the present work, a sample cohort of 100 volunteers, recruited from King’s College London and surrounding areas, was analyzed. The inclusion criteria for the patients were good general health, age between 20 and 70 years old, and body mass index (BMI) between 18.5 and 35 kg/m^2^. The average age of the patients was 34 years old, and the average BMI was 24.7 kg/m^2^. The exclusion criteria for the individuals included a history of cardiovascular disease, hypertension, diabetes, metabolic syndrome, terminal renal failure or malignancies, abnormal heart rhythm (between 60 and 100 bpm), allergy to berries, flaxseed or soy, smoking an irregular number of cigarettes, taking medications that can affect the cardiovascular system, recent loss of more than 10% of weight, pregnancy or planning to become pregnant in the next 6 months, and participation in another study in the past month.

Participants were asked to abstain from vegetables, fruits, wine, cocoa, chocolate, tea, and coffee 24 h prior to the first visit to reduce the influence of background diet. All subjects gave written informed consent before their participation in the study and agreed to maintain their eating and drinking habits and exercise habits for the duration of the study.

Subjects consumed a (poly)phenol intervention consisting of 30 g of milled flaxseeds (which contained 300 mg of lignans), 40 g of freeze-dried raspberry powder (containing 153 mg of ellagitannins), and 250 mL of soy milk (containing 22 mg of isoflavones) for 3 days. Spot urine samples were collected in a fasted state on day 1 (V1), and a 24 h urine sample was collected after consumption of the last breakfast on day 3 (V2). The urine sample volumes were recorded, and the samples were stored at −80 °C for subsequent analysis.

The study was conducted in accordance with the guidelines stated in the current revision of the Declaration of Helsinki, and informed consent was obtained for all subjects. All procedures involving human samples were approved by King’s College London Research Ethics Committee (HR-17/18-5353) and registered at the National Institutes of Health clinicaltrials.gov as NCT03573414.

### 2.2 Metabolite Extraction

The 200 samples were randomly divided into five batches of 40 samples each. Each batch was extracted separately, adhering to the same protocol. For the metabolite extraction, 50 μL of urine sample aliquots was utilized, and an isotopically labeled internal standard (IS) mixture, containing 5 μg/mL of tyrosine (^13^C_9_,^15^N), 10 μg/mL of phenylalanine (^13^C_9_,^15^N), and 20 μg/mL of valine (^13^C_5_), was spiked into each sample (10 μL). Subsequently, ice-cold LC-MS-grade methanol (4-fold) was added to the urine sample aliquots for protein precipitation. The solutions were vortexed and kept at −20 °C for 60 min. Afterward, they were centrifuged (Eppendorf, Centrifuge 5430 R) for 5 min at 4 °C and 14,000 rpm. The supernatants were collected and freeze-dried (Labconco, FreeZone 4.5 L Benchtop Freeze-Dryer) for 17 h. Finally, the freeze-dried pellet of all sample aliquots was resuspended with 50 μL of LC-MS grade acetonitrile:Milli-Q water (5:95) and vortexed for 15 s. Each supernatant was collected and transferred to an LC vial for UHPLC-MS/MS analysis. For the quality control sample (QC), 5 μL of each sample from all batches was pooled. The process remained the same for the QC sample, with the extraction and reconstitution volumes adjusted to its total volume. All 200 samples and QCs were analyzed via UHPLC-MS/MS with a randomized sample analysis list.

### 2.3 Enzymatic Treatment

For the enzymatic treatment, 15 μL each of 40 randomly selected samples, with an equal number from V1 and V2, was pooled and extracted following the protocol previously described in Section 2.2. The total sample volume was divided into four parts: two parts were utilized for treatment with the recombinant arylsulfatase ASPC (Kura Biotech, cat no. ASPC-10 mL, lot no. 6620) assay (control and treatment groups), and the other two parts for the recombinant β-glucuronidase B-One (Kura Biotech, B-One-10 mL, lot no. 1051) assay (details in the Supporting Information).

For the ASPC assay, the aliquots were reconstituted with Instant Buffer II (Kura Biotech, IB2-25 mL, lot no. 2514) and 1 U of ASPC was added to the treatment group to start the enzymatic reaction. The second aliquot was used as a control sample with the addition of denatured enzyme (30 min of incubation at 100 °C). The solutions were incubated at room temperature for 18 h (Thermomixer, 45 °C, 400 rpm). For the B-One assay, the aliquots were resuspended with 75 mM phosphate buffer, and the enzymatic reaction started with the addition of 100 U of B-One. Denatured enzyme (30 min of incubation at 100 °C) was added for the aliquot used as the control sample. The enzymatic treatment and control solutions were incubated at room temperature for 18 h (Thermomixer, 25 °C, 400 rpm). Aliquots were removed at 0 and 18 h, followed by addition of LC-MS-grade methanol (4-fold) for protein precipitation. All aliquots were dried in a SpeedVac Concentrator Plus System (Eppendorf, Germany), reconstituted with 5% acetonitrile:Milli-Q water (5:95), vortexed, and transferred to a suitable LC vial for analysis.

### 2.4 UHPLC-MS/MS Analysis

The UHPLC–MS/MS analysis was performed in a maXis II ETD Q-TOF mass spectrometer (Bruker Daltonics, Germany) using ESI as the source with an Elute UHPLC instrument (Bruker Daltonics, Germany). The separation was performed with an ACQUITY UPLC HSS T3 column (1.8 μm, 100 × 2.1 mm) from the Waters Corporation. For mobile phases, A contained Milli-Q water with 0.1% formic acid, and B contained LC–MS-grade methanol with 0.1% formic acid. The temperatures of the column and the autosampler were kept at 40 and 4 °C, respectively. The flow rate was set to 0.20 mL/min with an injection volume of 5 μL. The gradient used was as follows: 0–2 min, 0% B; 2–15 min, 0–100% B; 15–16 min, 100% B; 16–17 min, 100–0% B; 17–23 min, 0% B. The system was controlled using the Compass HyStar software package from Bruker (Bruker Daltonics, Germany). The MS acquisition was performed in negative ionization mode. The mass range was set from *m*/*z* = 50–1200. Data acquisition was performed in AutoMS/MS mode (data-dependent acquisition, DDA) with a cycle time of 0.5 s and a ramped collision energy from 20 to 50 eV. A solution of sodium formate [10 mM in a mixture of 2-propanol/water (1/1, v/v)] was used for internal calibration at the beginning of each LC-MS experiment in a segment between 0.10 and 0.31 min.

### 2.5 Data Analysis

The LC-MS raw data were converted into mzML format using MS convert (ProteoWizard) and were further processed with the XCMS package in R (version 4.3.2). For the enzymatic treatment data, an additional in-house R script was used to identify features with *m*/*z* differences of 79.9568 and 176.0321 Da for the identification of the sulfated and glucuronidated metabolites, respectively. The data set was filtered with the following criteria: features with retention time <1 min and >18 min and intensity level <10,000 total ion count were excluded. Then, the large data set was normalized in two steps. First, we applied SERRF (Systematic Error Removal using Random Forest), a QC-based method, to correct for variations that may arise from sample preparation or instrumental drift. Second, we normalized the data using creatinine levels as a reference to account for differences in sample concentrations across individuals (Supporting Information, Table S1).^12^

Statistical analysis was carried out in MetaboAnalyst 6.0 and GraphPad Prism 9.0 software (GraphPad Inc., San Diego, California, USA) by a two-tailed paired *t* test. *P*-values below <0.05 were considered significant, and box plots were generated.^13^ ROC analysis was performed in Python to evaluate the diagnostic performance of metabolite combinations by using binary logistic regression. For each combination of up to six metabolites, logistic regression models were fitted and the predicted probabilities were used to generate ROC curves. Combinations with an AUC greater than 0.75 were identified. The logistic regression model was validated with a 10-fold cross-validation analysis, yielding a mean AUC of 0.96 and a standard deviation of 0.03.

## 3 Results and Discussion

In this study, we performed an integrated metabolomics approach, combining semitargeted metabolomics with targeted enzymatic treatments to enhance the specificity and coverage of the metabolic profiling. We aimed at expanding the discovery of metabolites from these two important compound classes, elucidating their chemical structures, identifying potential biomarkers of (poly)phenol intake, and enhancing the understanding of their metabolic fate. We have previously reported the sulfatome analysis of 22 individuals from the same dietary intervention study, which led to the identification of 48 significantly upregulated sulfated metabolites, including 11 previously unknown sulfated metabolites.^10^ We have now expanded on the number of participants in the dietary intervention study (*n* = 100) and included the analysis of glucuronides, which will further enhance our understanding of phase II metabolic processes in the consumption of (poly)phenols.

### 3.1 Investigation of the Sulfated and Glucuronidated (Poly)phenol Metabolome

**Figure 2:**
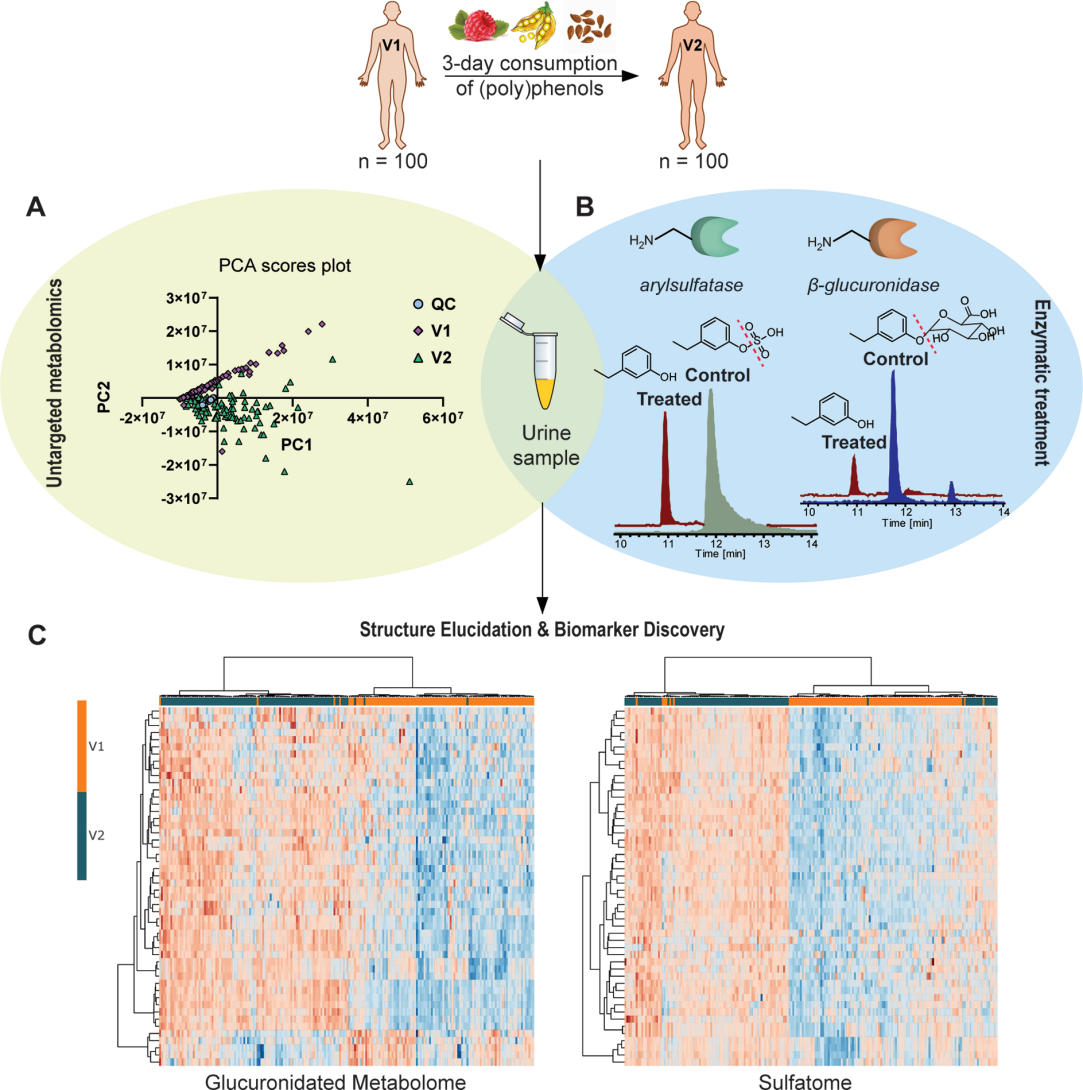
Workflow for the metabolomic analysis of urine samples collected from individuals (*N* = 100) before (V1) and after (V2) the consumption of a (poly)phenol-rich breakfast. (A) PCA score plot describing the global metabolomics investigation of the dietary intervention in combination with (B) enzymatic treatment methodology for the selective identification of sulfated and glucuronidated metabolites. Recombinant arylsulfatase ASPC (1 U) was used to hydrolyze sulfated, and recombinant β-glucuronidase B-One (100 U) was used to hydrolyze glucuronidated conjugates in pooled urine samples, allowing their targeted investigation by UHPLC-MS. (C) Heatmaps of the top 50 metabolites (paired *t* test) that describe the altered glucuronidated and sulfated metabolic profiles of the individuals. The heatmaps are generated with log_10_ transformed data.

As a first step, we performed an untargeted metabolomics approach to investigate global metabolic alterations before (V1) and after (V2) the consumption of a (poly)phenol-rich intervention. Unsupervised multivariate analysis (PCA) revealed a clear separation between the two groups, with the QC samples clustering together, demonstrating our high data quality and instrumental stability (Figure 2A). This variability is expected in metabolic studies and reflects individual responses to the dietary change possibly based on genetic differences, microbiota composition, and changes in the activity of phase II enzymes. Subsequently, we performed enzymatic treatment of pooled urine samples prior to LC-MS analysis, following our previously developed method^14,15^ (Figure 2B). To investigate sulfated metabolites, we utilized the recombinant arylsulfatase ASPC (1 U). Through bioinformatic analysis, searching for the difference of *m/z* = 79.9568 Da, we discovered 156 sulfated metabolites in the urine samples, most of which are in accordance with our previous analyses.^10,11^ The scope of our investigation was increased by inclusion of glucuronidated metabolites for the first time using the recombinant β-glucuronidase B-One (100 U). The bioinformatic search for features with a difference of *m*/*z* = 176.0321 Da, which corresponds to the glucuronic acid moiety, led to the detection of 143 glucuronidated metabolites in the same pooled urine samples. We then integrated the findings from the enzymatic treatment experiments with the global metabolomics data set, focusing on the alterations in glucuronidated and sulfated metabolites between V1 and V2 (Figure 2C). The heatmap of the top 50 metabolites, determined by a paired *t* test, reveals a clear clustering of the individuals in the baseline group (V1) and in the group after (poly)phenol consumption (V2).

### 3.2 Structure Elucidation of Sulfated and Glucuronidated Metabolites

**Figure 3:**
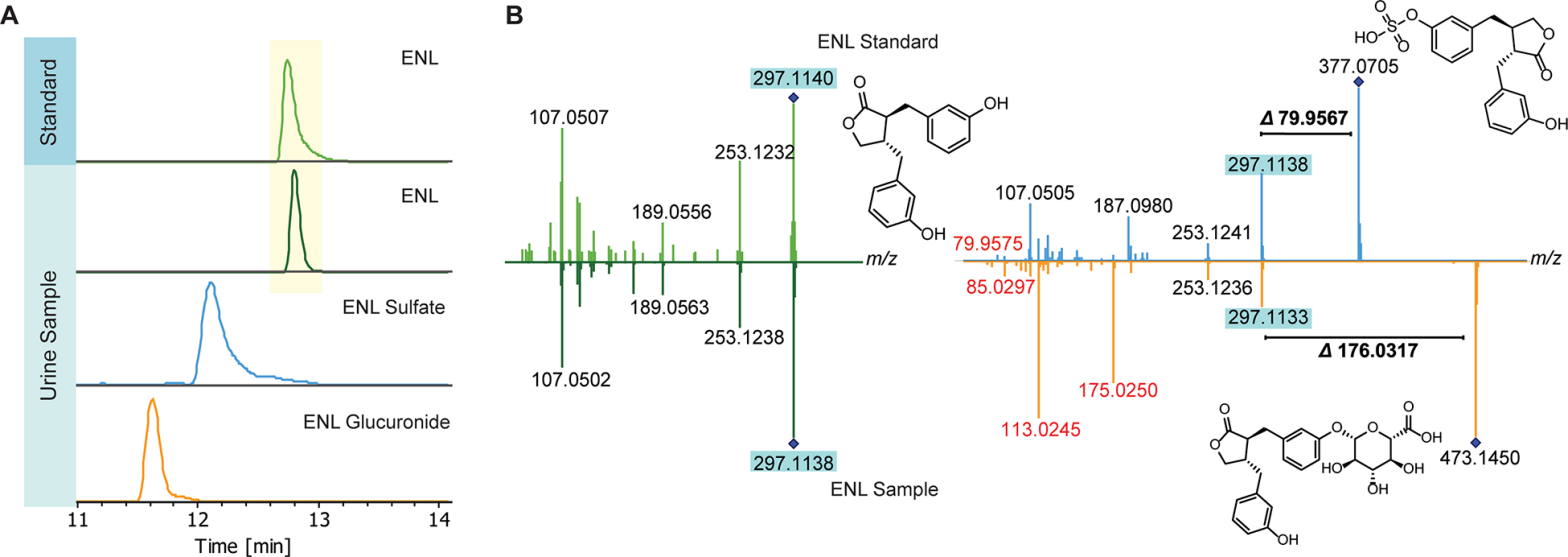
Structure validation of enterolactone (ENL) and its phase II modifications. (A) Chromatographic separation of all three metabolite forms and retention time match of natural ENL (*m*/*z* = 297.1138) and authentic standard. (B) Example for structure validation of ENL sulfate (*m*/*z* = 377.0705) and ENL glucuronide (*m*/*z* = 473.1450) based on the comparison of MS/MS spectra of ENL and its authentic standard (20–50 eV, negative ionization mode). The position of the sulfate and glucuronic acid groups is undetermined and shown in one of the possible positions.

Next, the putative phase II modifications were investigated to determine their chemical structures. We have successfully confirmed those structures by MS/MS fragmentation experiments, first to confirm the presence of the sulfate ester in 156 and glucuronic acid moiety in 143 metabolites, respectively (confidence level/CL 3/Supporting Information, Tables S2 and S3).^14,16^ We further validated the structure of 35 sulfates and 30 glucuronides in the urine samples at a higher level (CL 2), including non(poly)phenolic metabolites such as steroids. Validation at CL 2 was conducted using either MS/MS spectra from our in-house library or public libraries for the aglycon standard (CL 2a), or by comparing the MS/MS fragmentation patterns using computational tools such as SIRIUS (CL 2b).^17^ As an example, we illustrate the structure validation of enterolactone (ENL) and both of its phase II modifications using the reference standard of ENL (Figure 3A,B). Our enzymatic treatment methodology allows for the confirmation of the metabolite structure on the aglycon level, as previously described.^15^ Lastly, we obtained the highest level of confidence for eight sulfates and one glucuronide, confirming the metabolite structure by the synthesized or commercially available standards of our in-house library (CL 1).

### 3.3 Phase II Metabolite Modification Changes at an Individual Level

**Figure 4:**
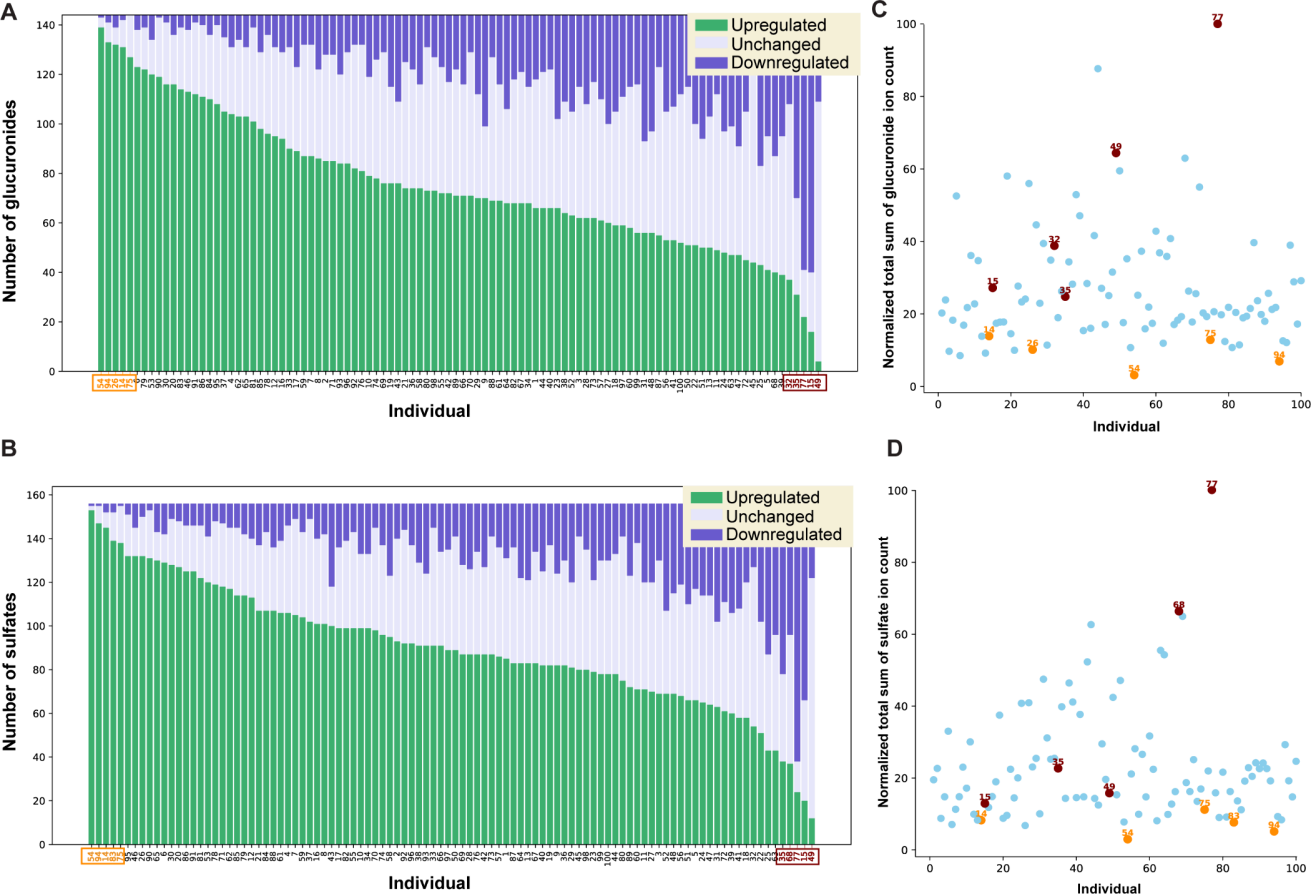
Investigation of the global phase II metabolome at an individual level. (A) Classification of individuals based on the number of glucuronides and (B) sulfates found upregulated (V2/V1 > 1.5). Downregulation is defined by V2/V1 < 0.67 and no change by 0.67 < V2/V1 < 1.5. (C) Scatter plot of the normalized total sum of glucuronide intensities and (D) normalized total sum of sulfate intensities at the baseline level for each individual. Each data point is represented as a percentage of the maximum observed intensity. (A–D) The individuals highlighted in orange are the top five individuals that exhibited either the highest production of glucuronides or sulfates after the intervention. The five individuals with the lowest production are highlighted in maroon.

After the comprehensive identification and structure elucidation of the glucuronidated and sulfated metabolites from the (poly)phenol-rich breakfast, we sought to investigate the variability in the total glucuronide and sulfate content across the entire cohort. The phase II metabolite production levels for a total of 143 glucuronidated and 156 sulfated metabolites were examined. We assessed the number of glucuronides that were upregulated, downregulated, or remained unchanged across all individuals, with subjects arranged in descending order based on the number of upregulated glucuronides (V2/V1 > 1.5) (Figure 4A). The same analysis was performed for the investigation of the corresponding entire sulfate production data set (Figure 4B). Interestingly, a substantial overlap was observed between the individuals that produce the glucuronide and sulfate conjugates (Supporting Information, Table S4). This high prevalence of glucuronidation and sulfation suggests that both phase II metabolic conversions of dietary (poly)phenols are consistent across individuals, as high sulfate producers are in most cases also high glucuronide producers and *vice versa*.

To further explore whether baseline metabolite levels have an influence on the upregulation patterns in V2, we assessed baseline glucuronide and sulfate levels across individuals. To compare the individual differences, we normalized according to the total sum of glucuronidated metabolite intensities (Figure 4C) and sulfated metabolite intensities (Figure 4D) at the baseline level for each individual. Each data point is represented as a percentage of the maximum observed intensity. To obtain a good and unbiased overview, we focused on the five highest and five lowest producers of glucuronides and sulfates. We observed that the five individuals with the highest total number of increased metabolite levels after the dietary intervention (V2/V1 > 1.5) were initially among the top 18 individuals with the lowest baseline levels of phase II metabolites (3–14%). Among the five lowest producers, one individual displayed the highest baseline levels for both phase II modifications. The other four individuals ranged between 13 and 66% demonstrating that these producers had diversely increased levels of phase II modifications and that the individual variability remains a major factor.

### 3.4 Discovery of Dietary Biomarkers

Univariate statistical analysis was performed in MetaboAnalyst and GraphPad Prism, where a paired *t* test revealed the significant alteration of 91 sulfated and 94 glucuronidated metabolites (*p*-value <0.05). The majority of the phase II metabolites were increased in the urine samples of the individuals after the consumption of the (poly)phenol breakfast (V2) compared to the baseline urine (V1) (Supporting Information, Tables S5 and S6). In the case of stereoisomers, when authentic reference standards of the sulfated and glucuronidated metabolites were unavailable, we have summed up the total ion count of the individual peaks. To ensure biological importance and statistical power, we have confirmed that all peaks were altered in the same way and that the MS fragmentation pattern confirms the metabolite core structure (Supporting Information, Figure S1). Among the investigated upregulated phase II metabolites are several isoflavones, such as daidzein and equol glucuronides, 4-ethylphenyl sulfate and glucuronide, demonstrating the efficient clearance of the dietary components. Elevated metabolites such as caffeic acid 3-sulfate, dihydrocaffeic acid sulfate, ferulic acid 4-*O*-sulfate, sinapic acid sulfate, and vanillin sulfate have been reported as downstream products of anthocyanin metabolism. Urolithins (urolithin A sulfate and urolithin A and B glucuronides) suggest the activity of the gut microbiota in converting ellagitannins after the consumption of raspberries. Lastly, the phase II conjugates of ENL, including ENL glucuronide and ENL sulfate, highlight the metabolism of dietary lignans, further reflecting on the role of the gut microbiota and phase II detoxification pathways in processing polyphenol-rich diets.

**Figure 5:**
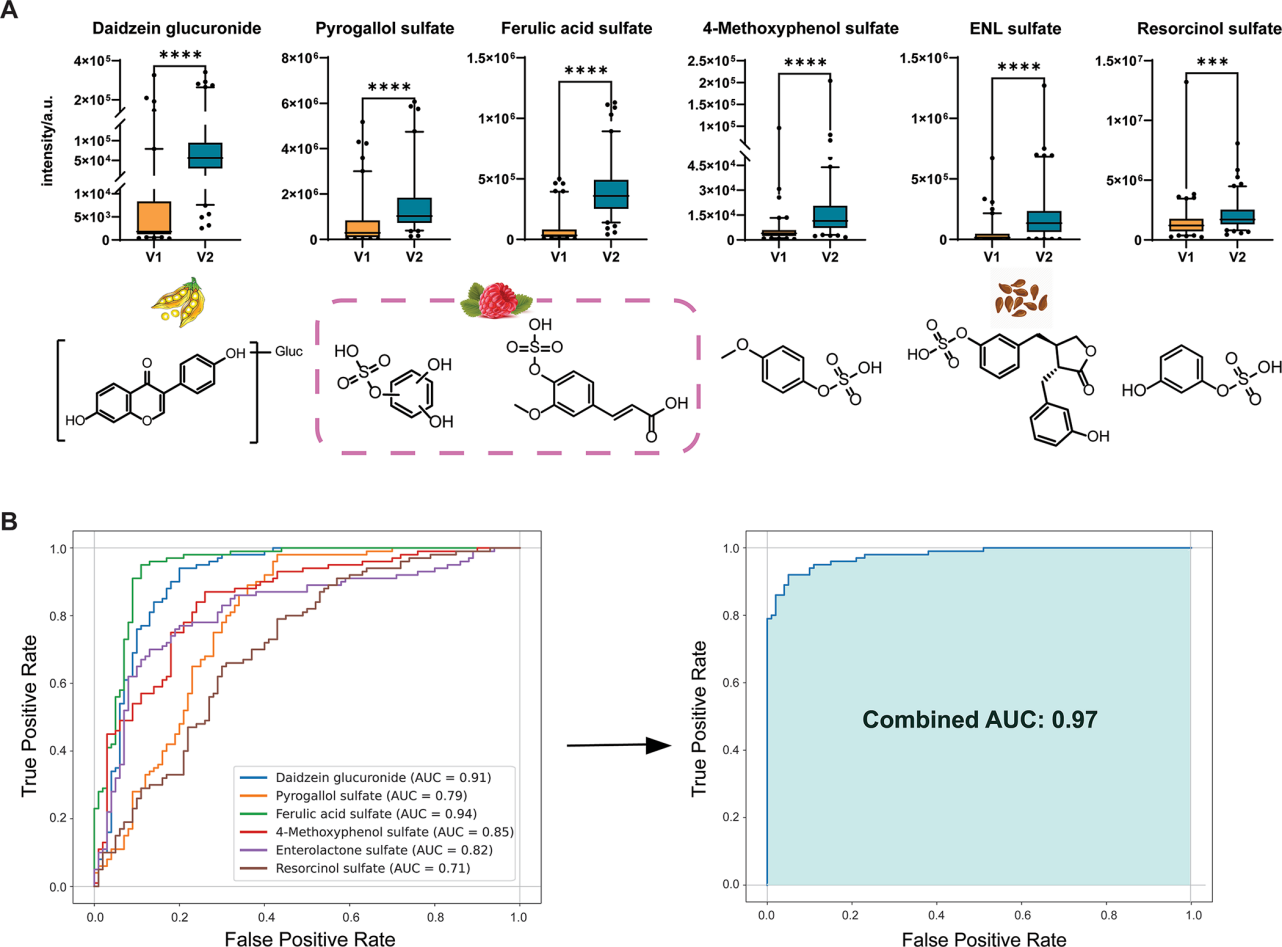
Selected metabolites identified as potential biomarkers of the (poly)phenol intervention. (A) Box plots (two-tailed paired *t* test, ****p ≤ 0.0001; ***p ≤ 0.001) and structures of the six conjugated metabolites with their main dietary source. The data points displayed fall outside the 95% confidence interval. (B) ROC curves for each individual metabolite with AUC values ranging from 0.71 to 0.94 were calculated using logistic regression models. The combined ROC curve was generated by evaluating the predictive performance of the combination of up to six metabolites with a significantly improved performance with an AUC of 0.97. AUC values were validated using 10-fold cross-validation to ensure robustness.

Receiver operating characteristic (ROC) analysis was performed to assess the diagnostic performance of different combinations of phase II metabolites derived from (poly)phenols. By evaluating the sensitivity and specificity of various combinations of metabolites, we can identify the most effective markers for dietary consumption. For this purpose, logistic regression models were used to generate ROC curves in Python for a combination of up to six metabolites of both sulfated and glucuronidated metabolites. The input for the ROC analysis consisted of 26 metabolites (10 glucuronidated and 16 sulfated metabolites) that were annotated at the highest confidence levels (Table S7). All possible combinations of metabolites were evaluated, calculating the area under the curve (AUC) for each combination. The final selection of the six key candidate biomarkers was determined by identifying the combination that yielded the highest AUC. The combination of daidzein glucuronide, pyrogallol sulfate, ferulic acid sulfate, 4-methoxyphenol sulfate, ENL sulfate, and resorcinol sulfate as the best-performing biomarkers demonstrates their potential significance in (poly)phenol dietary intervention. These metabolites were all significantly upregulated in V2 compared to V1 by the paired *t* test (Figure 5A). The ROC analysis of these individual metabolites yielded AUC values ranging from 0.71 to 0.94. In combination, these metabolites achieved outstanding predictive performance with an AUC of 0.97 (Figure 5B).

Daidzein glucuronide, an isoflavone conjugate metabolite, is one of the most abundant metabolites present in plasma after consumption of soy foods.^18^ It plays a role in hormone regulation and has been reported to prevent hormone-dependent cancer development.^19,20^ Pyrogallol is a major gut microbial metabolite of (poly)phenols, deriving from many different subclasses.^21−23^ It has been found in plasma and urine after the consumption of berries, and its sulfated conjugate is suggested to have similar properties to the parent compound, exhibiting anti-inflammatory and antimicrobial activity, hereby benefiting the gut and overall human health.^24−26^ Ferulic acid sulfate is a hydroxycinnamic acid derivative with evidence on cardiovascular health benefits.^23,27−30^ Ferulic acid is a natural component of plant cell walls and therefore is present in many plant foods such as wholegrain, fruits, vegetable, and coffee.^31^ Ferulic acid is also a metabolite derived from (poly)phenols and has been detected in plasma and urine samples after consumption of raspberries, which is the most likely source in the present study.^32^ 4-Methoxyphenol sulfate has been reported as a metabolite found in plasma and urine after the consumption of rice bran and navy bean powder.^33^ While little is known about its bioactivity, its upregulation has been associated with a reduction of tumor size in colorectal cancer models. This metabolite has not been reported as a compound known to be derived from any of the investigated diets (flaxseeds, raspberries, and soy). However, as our method identifies metabolites not commonly covered by standard methodologies and as this compound is upregulated after consumption of our dietary intervention, we hypothesize that it could be a breakdown product of food components present in the (poly)phenol-rich breakfast. ENL sulfate, a metabolite derived from lignans, has been reported and quantified in our previous study.^10^ Remarkably, a small meta-analysis of observational studies has linked ENL levels in plasma and urine with a lower CVD mortality risk.^34^ Another metabolite associated with several potential health benefits, such as antioxidant and anti-inflammatory properties, is resorcinol.^35,36^ However, research focusing on resorcinol sulfate remains limited. While the bioactivity of the majority of compounds is well established for the parent metabolites, the specific effects of their phase II modifications are largely unexplored. These findings suggest that the combined measurement of these metabolites serves as a robust biomarker panel for assessing the metabolic impact of (poly)phenol-rich interventions.

In summary, this study presents the first detailed investigation of the altered sulfated and glucuronidated metabolome in 100 individuals as a result of (poly)phenol-rich diet consumption. Our findings suggest the potential of nutrimetabolomics in identifying key (poly)phenol-derived metabolites as biomarkers for evaluating the effects of (poly)phenol-rich interventions. By investigating phase II biotransformations in human urine samples at a global level, we provide a comprehensive understanding of changes in these metabolic processes that affect the biological activity of (poly)phenols, hence contributing to the development of dietary strategies that maximize the health benefits of (poly)phenolic intake. We identified a panel of six metabolites that provided exceptional predictive accuracy in an ROC analysis for the dietary intake with a combined AUC of 0.97. These candidate biomarkers have potential applications in nutritional research by providing a more accurate assessment of (poly)phenol intake, which is often estimated through self-reported dietary questionnaires and can be unreliable. Additionally, this metabolite panel can assist in identifying individuals with low (poly)phenol intake that could lead to personalized dietary recommendations to enhance potential health benefits. Although further validation in independent cohorts is required, our findings provide a solid and novel foundation for improved dietary monitoring. Furthermore, the classification of individuals based on their capacity to metabolize (poly)phenols into glucuronidated and sulfated metabolites may provide new insights into the individual variability in metabolic responses to (poly)phenol consumption. These differences can be influenced by relevant factors such as the gut microbiota composition, the activity of UGT and SULT enzymes, and excretion rates. As these conjugation processes facilitate the clearance and excretion of bioactive metabolites, our results may demonstrate the potential at an individual level to identify personalized nutritional benefits of (poly)phenol consumption that can be applied to other nutritional studies, as well. Future studies of poly(phenol)-rich diets and disease-related investigations can utilize our developed tools and screen for the identified novel metabolites described in our study.

## Supporting Information Available

Urine creatinine concentrations (Table S1), validated sulfated and glucuronidated metabolites (Tables S2 and S3), individual variations in metabolite conversions (Table S4), significantly altered sulfated and glucuronidated metabolites (Tables S5 and S6), input metabolites for ROC analysis (Table S7), additional figure (Figure S1) with examples of isomeric metabolites, and experimental procedure details on the determination of glucuronidase and arylsulfatase activities (PDF)
