# Supplementary material for: Characterizing the Sulfated and Glucuronidated (Poly)phenol Metabolome for Dietary Biomarker Discovery
Source: J Agric Food Chem. 2025 Mar 3;73(11):6702–10. doi: 10.1021/acs.jafc.4c12596 (PMC11926854; doi:10.1021/acs.jafc.4c12596)
Supplement: Supplementary file 1 — jf4c12596_si_001.pdf [file jf4c12596_si_001.pdf]

Supporting Information

Characterizing the Sulfated and Glucuronidated (Poly)phenol Metabolome for Dietary Biomarker Discovery

Ioanna Tsiara [a], Belén Hervás Povo [a], Wafa Alotaibi [b], Paul Young Tie Yang [b], Ana Rodriguez-Mateos [b], Daniel Globisch [a*]

[a] Department of Chemistry - BMC, Science for Life Laboratory, Uppsala University, Box 576, SE-75124, Uppsala, Sweden

[b] Department of Nutritional Sciences, School of Life Course and Population Sciences, Faculty of Life Sciences and Medicine, King's College London, UK

Table of contents

Figures 2
Tables..3
General..29
Description of procedures..29
Determination of glucuronidase activity 29
Determination of arylsulfatase activity 30

Figures

A

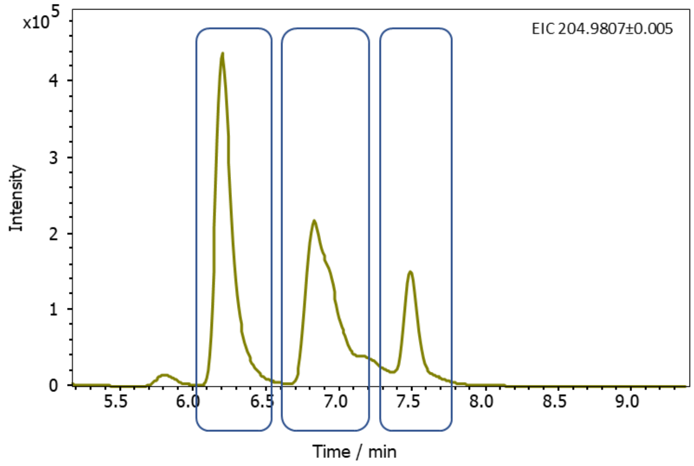

B

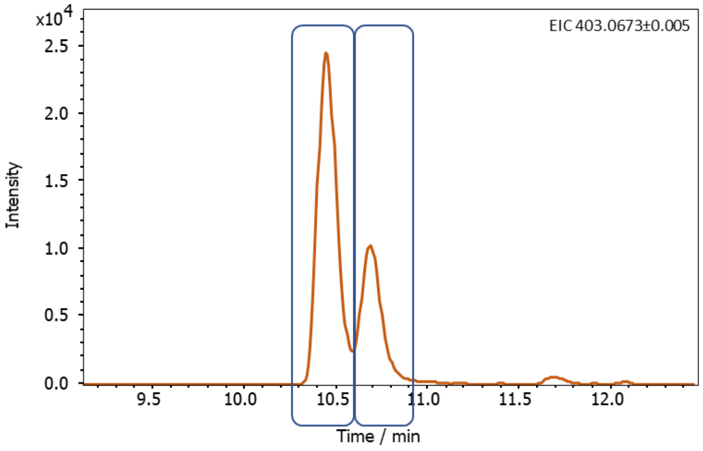

Figure S1. Examples of isomeric metabolites. (A) Pyrogallol sulfate (*m/z* = 204.9807) and (B) urolithin-A-glucuronide (*m/z* = 403.0673). The individual metabolite peaks were summed-up for the investigation of candidate biomarkers.

Tables

Table S1. Creatinine concentrations in spot urine samples of volunteers before (V1) and 24 h collection of urine samples after consumption of a (poly)phenol rich breakfast (V2) for 3 days.

Name	Ucrea (mmol/L)	Name	Ucrea (mmol/L)
V1-SS048	5.851	V2-SS048	3.792
V1-SS049	13.695	V2-SS049	10.187
V1-SS050	6.132	V2-SS050	4.852
V1-SS051	5.563	V2-SS051	4.568
V1-SS052	22.326	V2-SS052	10.780
V1-SS053	17.598	V2-SS053	5.296
V1-SS054	36.766	V2-SS054	4.090
V1-SS055	6.768	V2-SS055	6.712
V1-SS056	10.134	V2-SS056	4.486
V1-SS057	3.848	V2-SS057	1.294
V1-SS058	5.658	V2-SS058	3.262
V1-SS059	8.396	V2-SS059	6.608
V1-SS060	8.560	V2-SS060	3.416
V1-SS061	8.440	V2-SS061	8.316
V1-SS062	10.463	V2-SS062	2.280
V1-SS063	14.306	V2-SS063	4.950
V1-SS064	13.395	V2-SS064	5.840
V1-SS066	18.277	V2-SS066	5.344
V1-SS067	12.832	V2-SS067	14.024
V1-SS069	29.881	V2-SS069	15.398
V1-SS070	7.821	V2-SS070	8.256
V1-SS071	29.091	V2-SS071	7.368
V1-SS072	10.866	V2-SS072	5.344
V1-SS073	2.652	V2-SS073	7.362
V1-SS074	4.266	V2-SS074	3.916
V1-SS075	20.450	V2-SS075	3.434
V1-SS076	11.736	V2-SS076	8.904
V1-SS077	12.948	V2-SS077	12.554
V1-SS079	4.724	V2-SS079	3.294
V1-SS080	11.118	V2-SS080	3.298
V1-SS081	7.706	V2-SS081	4.940
V1-SS082	1.258	V2-SS082	2.700
V1-SS083	1.704	V2-SS083	4.004
V1-SS084	10.204	V2-SS084	9.430
V1-SS085	13.334	V2-SS085	14.558
V1-SS086	17.340	V2-SS086	2.630

V1-SS087	0.978	V2-SS087	3.092
V1-SS088	2.064	V2-SS088	2.688
V1-SS089	31.752	V2-SS089	10.824
V1-SS090	4.540	V2-SS090	3.116
V1-SS091	3.874	V2-SS091	3.424
V1-SS092	12.266	V2-SS092	6.504
V1-SS093	3.392	V2-SS093	4.800
V1-SS094	10.718	V2-SS094	15.226
V1-SS095	2.114	V2-SS095	7.548
V1-SS096	9.088	V2-SS096	4.934
V1-SS097	28.616	V2-SS097	4.768
V1-SS098	19.604	V2-SS098	3.280
V1-SS099	3.098	V2-SS099	5.006
V1-SS101	11.18	V2-SS101	18.450
V1-SS102	2.644	V2-SS102	1.474
V1-SS103	5.792	V2-SS103	4.424
V1-SS104	6.150	V2-SS104	10.566
V1-SS105	5.172	V2-SS105	12.688
V1-SS106	15.382	V2-SS106	4.030
V1-SS107	11.336	V2-SS107	9.740
V1-SS108	2.690	V2-SS108	3.646
V1-SS109	0.916	V2-SS109	4.302
V1-SS110	16.760	V2-SS110	8.636
V1-SS112	2.452	V2-SS112	3.038
V1-SS113	12.038	V2-SS113	9.978
V1-SS115	2.434	V2-SS115	2.960
V1-SS116	1.508	V2-SS116	3.502
V1-SS117	14.694	V2-SS117	4.392
V1-SS118	4.646	V2-SS118	3.124
V1-SS119	4.144	V2-SS119	12.702
V1-SS120	4.132	V2-SS120	2.154
V1-SS121	6.548	V2-SS121	6.220
V1-SS122	4.204	V2-SS122	6.188
V1-SS124	2.170	V2-SS124	6.854
V1-SS125	15.780	V2-SS125	4.966
V1-SS126	4.668	V2-SS126	6.414
V1-SS127	4.184	V2-SS127	9.866
V1-SS128	2.126	V2-SS128	5.346
V1-SS129	17.804	V2-SS129	3.776
V1-SS130	2.158	V2-SS130	5.988
V1-SS131	4.408	V2-SS131	9.198

V1-SS132	14.626	V2-SS132	11.238
V1-SS133	3.460	V2-SS133	8.034
V1-SS134	24.378	V2-SS134	14.970
V1-SS135	18.394	V2-SS135	3.846
V1-SS136	1.602	V2-SS136	2.920
V1-SS137	12.288	V2-SS137	12.352
V1-SS138	9.486	V2-SS138	11.656
V1-SS139	21.404	V2-SS139	9.418
V1-SS140	13.006	V2-SS140	7.602
V1-SS141	13.156	V2-SS141	1.918
V1-SS142	26.298	V2-SS142	31.266
V1-SS143	19.000	V2-SS143	6.392
V1-SS144	4.088	V2-SS144	4.930
V1-SS146	3.928	V2-SS146	4.284
V1-SS147	2.228	V2-SS147	3.564
V1-SS148	10.074	V2-SS148	11.230
V1-SS149	16.850	V2-SS149	9.766
V1-SS150	28.112	V2-SS150	4.296
V1-SS152	6.282	V2-SS152	7.566
V1-SS153	6.522	V2-SS153	2.724
V1-SS154	23.572	V2-SS154	21.356
V1-SS155	4.664	V2-SS155	3.838
V1-SS157	8.118	V2-SS157	7.784

Table S2. All validated sulfated metabolites with annotated confidence levels.

(Level 1: Validation with authentic synthetic or commercial standards; Level 2a: Metabolite structure validation based on unambiguous matching of MS^2 spectra with experimental spectra from literature or library sources; Level 2b: Identification of the molecular formula and MS^2 fragmentation pattern comparison using computational tools; Level 3: MS^2-validation of glucuronic acid moiety in the metabolite).

#	m/z	Rt / min	Chemical formula	CL
1	172.9915	8.17	C6H5O4S-	1
2	182.9972	3.12	-	3
3	187.0074	10.23	C7H7O4S-	3
4	188.0026	5.33	C6H8NO4S-	3
5	188.0104	10.17	C6H5NO4S-	2b
6	188.9863	7.52	C6H5O5S-	2b
7	188.9865	5.42	C6H5O5S-	3
8	192.9814	5.62	-	3
9	199.0073	11.30	C8H7O4S-	3
10	201.0228	11.97	C8H9O4S-	2b
11	203.0020	9.63	C7H7O5S-	1
12	203.0020	8.70	C7H7O5S-	2b
13	203.0022	6.80	C7H7O5S-	3
14	203.0022	7.09	C7H7O5S-	1
15	203.9976	7.97	C6H6NO5S-	3
16	204.9814	6.24	C6H5O6S-	2b
17	204.9814	6.83	C6H5O6S-	2b
18	204.9814	7.43	C6H5O6S-	2b
19	210.0410	8.82	-	3
20	212.0024	8.48	C8H6NO4S-	2a
21	212.0024	9.20	C8H6NO4S-	3
22	212.0025	7.97	C8H6NO4S-	3
23	213.0237	7.46	C9H9O4S-	2b
24	215.0021	8.48	C8H7O5S-	3
25	215.0022	9.08	C8H7O5S-	2b
26	215.0023	10.85	C8H7O5S-	3
27	216.0339	6.30	C8H10NO4S-	2b
28	216.9813	7.62	C7H5O6S-	2b
29	216.9813	8.23	C7H5O6S-	2b
30	217.0178	11.47	C6H5O6S-	2b
31	217.0305	1.80	-	3
32	218.0495	6.50	-	3
33	218.0495	6.80	-	3
34	218.9970	6.38	C7H7O6S-	3

35	218.9970	7.40	C7H7O6S-	2b
36	218.9970	8.35	C7H7O6S-	3
37	218.9971	6.78	C7H7O6S-	3
38	220.9764	6.57	C6H5O7S-	3
39	220.9764	7.12	C6H5O7S-	3
40	221.0127	8.88	C7H9O6S-	3
41	225.0631	5.72	-	3
42	227.9972	7.02	C8H6NO5S-	3
43	227.9973	7.63	C8H6NO5S-	3
44	227.9974	8.00	C8H6NO5S-	3
45	228.9938	2.12	-	3
46	229.0178	11.33	C9H9O5S-	3
47	229.0178	10.62	C9H9O5S-	2b
48	229.0178	12.07	C9H9O5S-	2b
49	229.0542	14.35	C10H13O4S-	2b
50	230.0130	7.20	C8H8NO5S-	3
51	230.0130	8.57	C8H8NO5S-	3
52	230.0131	9.57	C8H8NO5S-	3
53	230.9971	8.70	C8H7O6S-	1
54	230.9972	7.00	C8H7O6S-	3
55	230.9972	10.08	C8H7O6S-	3
56	232.9764	7.49	C7H5O7S-	2b
57	232.9764	7.22	C7H5O7S-	2b
58	233.0127	8.68	C8H9O6S-	3
59	233.0127	7.20	C8H9O6S-	3
60	233.0127	9.25	C8H9O6S-	3
61	233.0127	7.72	C8H9O6S-	3
62	239.0597	10.58	-	3
63	240.9815	9.07	C9H4O6S-	3
64	242.0130	7.98	C9H8NO5S-	3
65	242.0131	9.57	C9H8NO5S-	3
66	242.0131	8.85	C9H8NO5S-	3
67	242.9964	9.13	C9H7O6S-	1
68	242.9971	9.42	C9H7O6S-	3
69	242.9971	9.73	C9H7O6S-	3
70	243.0334	12.48	C10H11O5S-	3
71	243.9923	6.22	C8H6NO6S-	3
72	243.9924	7.03	C8H6NO6S-	3
73	245.0125	9.27	C9H9O6S-	3
74	245.0492	10.65	C10H13O5S-	2b
75	246.0080	6.93	C8H8NO6S-	3

76	246.9919	7.00	C8H7O7S-	2b
77	246.9919	8.02	C8H7O7S-	2b
78	246.9919	7.77	C8H7O7S-	2b
79	247.0283	8.78	C9H11O6S-	3
80	249.0074	6.96	C8H9O7S-	2b
81	249.0077	4.33	C8H9O7S-	3
82	249.0077	6.62	C8H9O7S-	2b
83	249.0077	7.49	C8H9O7S-	2b
84	258.0080	7.35	C9H8NO6S-	3
85	258.9919	9.20	C9H5O7S-	2b
86	258.9920	8.88	C9H5O7S-	3
87	258.9921	8.58	C9H5O7S-	3
88	259.0283	9.45	C10H10O6S-	3
89	259.0284	12.35	C10H10O6S-	3
90	260.0234	5.75	C9H10NO6S-	2b
91	260.0237	9.00	C9H10NO6S-	3
92	261.0074	8.73	C9H9O7S-	1
93	261.0076	7.88	C9H9O7S-	3
94	261.0440	9.95	-	3
95	262.9869	7.83	C9H10NO6S-	2b
96	263.0047	8.73	C9H7O6S-	3
97	263.0232	5.73	C9H11O7S-	3
98	263.0233	7.15	C9H11O7S-	3
99	263.0233	9.52	C9H11O7S-	3
100	266.9641	10.15	C7H6O7S2-	3
101	271.0284	9.43	C11H11O6S-	3
102	272.9930	2.62	C6H9O10S-	3
103	273.0075	9.28	C10H9O7S-	1
104	274.0028	7.25	C9H8NO7S-	3
105	274.0393	8.08	C10H12NO6S-	3
106	275.0233	9.47	C10H11O7S-	2b
107	275.0711	6.95	-	3
108	275.0711	6.07	-	3
109	276.0550	7.13	C10H14NO6S-	3
110	277.0026	7.93	C9H9O8S-	2b
111	287.0230	9.05	C11H11O7S-	2b
112	289.0029	9.18	-	3
113	289.0381	9.12	C11H13O7S-	3
114	300.0186	8.92	C11H10NO7S-	3
115	303.0182	9.12	C11H11O8S-	1
116	303.0296	5.93	-	3

117	303.0723	7.88	-	3
118	303.0724	7.48	-	3
119	305.0336	8.67	C11H13O8S-	3
120	305.0339	9.00	C11H13O8S-	3
121	305.0475	6.20	C11H13O8S-	2b
122	306.9921	11.53	C13H7O7S-	3
123	309.0554	8.97	-	3
124	319.0673	5.95	C10H13N2O5S-	3
125	320.0624	5.63	-	3
126	325.0761	8.27	-	3
127	330.0291	8.70	C12H12NO8S-	3
128	333.0077	11.38	C15H9O7S-	3
129	336.0728	9.37	-	3
130	341.1244	13.45	-	3
131	343.0672	7.92	-	3
132	351.0571	2.67	C16H15O7S-	3
133	352.0860	13.28	C16H18NO6S-	3
134	352.0939	9.93	-	3
135	354.1019	12.88	-	3
136	356.1174	13.20	C16H22NO6S-	3
137	372.1122	10.72	C16H22NO7S-	3
139	373.0463	7.29	-	3
140	377.0702	12.20	C18H17O7S-	2b
141	381.1015	11.85	C18H21O7S-	3
142	383.0445	9.80	-	3
143	383.1925	9.83	-	3
144	387.2026	11.91	-	3
145	397.1142	9.08	-	3
146	408.0620	8.42	-	3
147	411.1297	11.98	-	3
148	421.0600	13.28	-	3
149	423.0758	11.42	C18H17O7S-	3
150	425.0121	8.47	-	3
151	433.2081	13.82	C24H33O5S-	3
152	441.1227	10.87	-	3
153	447.0935	11.40	-	3
154	447.0936	10.57	-	3
155	459.1509	12.07	-	3
156	517.1353	12.63	-	3

Table S3. All validated glucuronidated metabolites with annotated confidence levels.

(Level 1: Validation with authentic synthetic or commercial standards; Level 2a: Metabolite structure validation based on unambiguous matching of MS^2 spectra with experimental spectra from literature or library sources; Level 2b: Identification of the molecular formula and MS^2 fragmentation pattern comparison using computational tools; Level 3: MS^2-validation of glucuronic acid moiety in the metabolite).

#	m/z	RT / min	Chemical formula	CL
1	269.0669	8.80	C12H13O7-	3
2	283.0825	10.37	C13H15O7-	1
3	285.0618	8.60	C12H13O8-	3
4	287.0774	7.32	-	3
5	297.0982	11.70	C14H17O7-	2b
6	297.0984	12.88	C14H17O7-	2b
7	301.0567	7.22	C12H13O9-	2b
8	301.0568	8.30	C12H13O9-	3
9	303.0723	7.48	C27H27O16-	3
10	303.0724	7.88	-	3
11	306.1196	8.26	-	3
12	308.0779	7.48	C14H14NO7-	2b
13	317.0517	6.01	-	3
14	317.1244	12.70	-	3
15	319.0672	5.95	C14H17O7-	3
16	319.0673	6.20	-	3
17	319.0673	6.38	-	3
18	319.1400	13.68	C14H23O8-	3
19	319.1401	13.33	C14H23O8-	3
20	324.0725	8.27	C14H14NO8-	3
21	324.0728	7.27	C14H14NO8-	3
22	325.0933	11.75	C15H17O8-	2b
23	325.1295	13.50	-	3
24	326.0883	6.73	C14H16NO8-	3
25	326.0885	8.90	C14H16NO8-	3
26	327.1089	12.48	-	3
27	328.0677	6.92	C13H14NO9-	2b
28	329.1608	14.18	-	3
29	331.1401	13.50	-	3
30	335.1350	11.77	C14H23O9-	3
31	338.0884	7.27	C15H16NO8-	3
32	339.1088	12.15	C16H19O8-	2b
33	339.1088	12.92	C16H19O8-	3
34	340.0676	5.68	C14H15NO9	3

35	341.0880	9.32	C15H17O9-	2b
36	341.1244	11.05	C16H21O8-	2b
37	341.1244	13.07	C16H21O8-	2b
38	341.1245	13.45	-	3
39	343.0672	7.92	C14H15O10-	3
40	343.0673	7.50	C14H15O10-	3
41	343.1400	13.47	C16H23O8-	3
42	343.1401	13.97	C16H23O8-	3
43	343.1402	14.32	C16H23O8-	3
44	345.1556	10.83	C16H25O8-	3
45	345.1557	11.28	C16H25O8-	3
46	345.1557	11.83	C16H25O8-	3
47	345.1558	12.25	-	3
48	345.1558	12.48	-	3
49	345.1558	12.75	-	3
50	345.1558	14.25	-	3
51	347.1714	13.38	-	3
52	349.1142	9.78	C14H21O10-	3
53	350.0883	9.90	C16H16NO8-	2b
54	355.0898	7.42	-	3
55	355.1038	12.37	C16H19O9-	2b
56	357.0828	7.55	C15H17O10-	3
57	357.0830	8.32	C15H17O10-	3
58	358.1332	14.09	-	3
59	359.0987	5.83	C15H19O10-	3
60	359.1350	11.05	C16H23O9-	3
61	359.1715	15.09	-	3
62	361.1506	11.85	C16H25O9-	3
63	361.1506	12.12	C16H25O9-	3
64	361.1506	12.52	C16H25O9-	3
65	363.1662	12.15	-	3
66	367.1038	9.35	C17H19O9-	3
67	369.0829	9.90	C16H17O10-	2b
68	369.0830	8.72	C16H17O10-	2b
69	369.0832	10.87	C16H17O10-	2b
70	369.1194	11.33	C17H21O9-	3
71	371.0985	9.70	C16H19O10-	2b
72	371.0986	9.13	C16H19O10-	2b
73	371.0986	9.38	C16H19O10-	2b
74	375.1300	11.22	C16H22O10-	3
75	381.1556	14.68	C19H26O8	2b

76	383.0985	9.07	C17H19O10-	2b
77	383.0986	8.75	C17H19O10-	3
78	383.1194	5.88	-	3
79	383.1197	6.55	-	3
80	385.1507	12.08	C16H23N3O8-	3
81	387.0723	11.74	C19H15O9-	3
82	387.1663	12.87	C18H27O9-	3
83	387.2026	11.90	-	3
84	389.0990	8.07	-	3
85	389.1456	12.08	-	3
86	389.1818	13.08	C18H29O9-	3
87	394.1357	7.22	-	3
88	397.1142	9.08	C18H21O10-	3
89	397.1505	12.03	-	3
90	397.1505	12.57	-	3
91	401.1818	9.75	-	3
92	403.0673	10.42	C19H15O10-	3
93	403.0673	10.66	C19H15O10-	3
94	403.1976	9.02	-	3
95	403.1976	9.53	-	3
96	405.1767	12.43	-	3
97	411.1297	11.98	C19H23O10-	3
98	415.1976	13.82	-	3
99	415.1976	14.10	-	3
100	417.1193	11.33	C21H21O9-	2b
101	417.2132	13.03	-	3
102	419.2289	15.40	-	3
103	425.1456	11.73	C20H25O10-	2b
104	425.1819	12.35	C21H29O9-	3
105	425.1820	13.43	C21H29O9-	3
106	427.1976	13.85	-	3
107	429.0830	10.92	C21H17O10-	2b
108	429.0830	10.06	C21H17O10-	2b
109	429.1769	13.65	-	3
110	429.1770	13.73	-	3
111	429.1770	13.90	-	3
112	431.1923	13.17	C23H29NO7-	3
113	431.2286	13.28	-	3
114	431.2288	13.67	-	3
115	433.1142	12.17	C21H21O10-	2b
116	433.2081	13.48	C20H33O10-	3

117	433.2081	13.82	C20H33O10-	3
118	435.2238	14.40	-	3
119	439.1611	12.42	C21H27O10-	3
120	439.1612	10.95	C21H27O10-	3
121	443.1559	14.73	-	3
122	450.1772	11.98	C22H28NO9-	3
123	452.1927	12.35	-	3
124	452.1927	12.63	-	3
125	453.1768	12.85	C22H29O10-	3
126	459.1510	12.07	-	3
127	461.2394	14.67	-	3
128	463.2341	14.84	C25H35O8-	2b
129	465.2493	15.15	C25H37O8-	2a
130	465.2496	15.66	C25H37O8-	2b
131	473.1452	11.64	C24H25O10-	2b
132	477.1766	11.35	C24H29O10-	3
133	481.2443	13.68	C25H37O9-	3
134	489.2707	15.57	C24H41O10-	3
135	495.2238	14.50	C25H35O10-	3
136	495.2238	11.25	C25H35O10-	3
137	505.2656	14.10	C24H41O11-	3
138	507.2086	13.57	-	3
139	511.2550	14.02	-	3
140	539.2497	13.98	C27H39O11-	3
141	539.2498	13.50	C27H39O11-	3
142	541.2653	14.05	C27H41O11-	2b
143	640.3340	13.12	-	3

Table S4. Comparison of the total number of glucuronidated and sulfated metabolite conversions per individual. The table presents the number of glucuronides and sulfates that are upregulated (V2/V1 > 1.5), downregulated (V2/V1 < 0.67), or remained unchanged (0.67 ≤ V2/V1 ≤ 1.5) across the 100 individuals, arranged in descending order based on the upregulation of glucuronidated metabolites.

	**Glucuronides**			**Sulfates**		
# Individual	Upregulated	Downregulated	Unchanged	Upregulated	Downregulated	Unchanged
54	138	1	4	153	1	2
94	132	3	8	147	1	8
26	131	5	7	132	6	518
14	130	2	11	145	4	7
75	126	0	17	138	1	17
6	122	6	15	129	14	13
79	121	5	17	115	14	27
53	119	10	14	120	15	21
90	118	1	24	131	3	22
30	115	3	25	128	7	21
20	115	8	20	127	8	21
83	113	5	25	140	4	12
46	112	6	25	132	11	13
91	111	3	29	125	10	21
86	110	4	29	125	10	21
84	109	8	26	107	13	36
95	107	4	32	132	5	19
37	104	9	30	102	7	47
4	103	13	27	106	10	40
65	102	13	28	130	13	13
62	102	10	31	117	11	28
81	100	5	38	122	10	24
85	97	15	31	115	11	30
78	95	13	35	119	8	29
12	95	8	40	113	16	27
16	93	15	35	101	16	39
33	90	13	40	91	11	54
17	88	21	34	99	20	37
59	86	12	45	104	13	39
7	86	12	45	105	7	44
8	85	22	36	101	14	41
71	84	16	43	118	9	29
2	84	16	43	93	16	47
96	83	15	45	92	19	45
93	83	24	36	91	32	33

92	81	12	50	92	12	52
76	81	12	50	87	22	47
10	78	25	40	100	23	33
74	77	18	48	98	19	39
69	75	35	33	89	28	39
43	75	15	53	100	38	18
19	75	29	39	82	19	55
21	74	22	47	107	19	30
58	73	17	53	95	33	28
36	73	13	57	82	26	48
98	73	28	42	80	21	55
80	73	19	51	75	15	66
55	72	27	44	99	13	44
42	72	21	50	87	29	40
89	71	21	51	72	33	51
70	70	28	45	98	11	47
66	70	15	58	92	22	42
29	69	45	29	81	36	39
9	69	32	42	82	32	42
88	68	17	58	107	20	29
61	68	28	47	106	17	33
82	68	26	49	100	17	39
67	67	23	53	83	22	51
64	67	29	47	84	34	38
34	67	38	38	99	23	34
44	66	22	55	78	23	55
40	65	26	52	82	31	43
23	65	23	55	79	35	42
1	65	42	36	85	25	46
38	63	39	41	91	27	38
52	63	35	45	69	49	38
73	62	29	52	87	15	54
28	62	36	45	87	30	39
3	61	27	55	69	26	61
57	60	34	49	86	20	50
27	60	44	39	70	36	50
97	59	39	45	89	21	46
18	59	33	51	58	36	62
60	58	29	56	71	18	67
99	56	28	59	78	26	52
48	55	21	67	69	41	46

31	55	51	37	64	54	38
87	55	47	41	83	20	53
56	53	39	51	68	37	51
41	52	32	59	58	48	50
100	52	37	54	78	26	52
50	51	44	48	89	15	52
22	50	49	44	51	54	51
51	50	29	64	66	46	44
13	49	41	53	83	35	38
11	49	32	62	71	36	49
24	48	46	49	65	42	49
63	47	45	51	43	60	53
47	47	53	43	64	42	50
72	44	17	82	61	45	50
45	44	39	60	80	15	61
25	43	61	39	43	69	44
5	41	48	54	66	39	51
68	40	57	46	37	60	59
39	38	49	56	60	50	46
32	37	36	70	54	29	73
35	31	73	39	38	78	40
77	21	103	19	24	118	14
15	16	103	24	20	90	46
49	4	34	105	12	34	110

Table S5. All significantly altered sulfated metabolites with annotated confidence levels.

(Level 1: Validation with authentic synthetic or commercial standards; Level 2a: Metabolite structure validation based on unambiguous matching of MS^2 spectra with experimental spectra from literature or library sources; Level 2b: Identification of the molecular formula and MS^2 fragmentation pattern comparison using computational tools; Level 3: MS^2-validation of sulfate ester moiety in the metabolite). *the sum of the peaks has been used for investigation of candidate biomarkers. ↓ marks downregulation at V2 compared to V1.

#	Name	Structure	Chemical formula	m/z Experimental	m/z Theoretical	ppm difference	RT /min	Level of confidence	*P*-value
1	Phenol sulfate	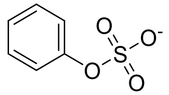	$C_6H_5O_4S^-$	172.9915	172.9914	-0.44	8.16	1	0.0367
2	Resorcinol sulfate	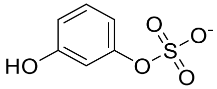	$C_6H_5O_5S^-$	188.9863	188.9863	-0.24	7.52	2b	0.000319
3	-	-	-	192.9814	-	-	5.62	3	<0.000001
4	4-Ethylphenylsulfate	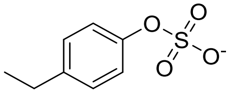	$C_8H_9O_4S^-$	201.0228	201.0227	-0.73	11.97	2b	0.000003
5	4-methoxyphenol sulfate	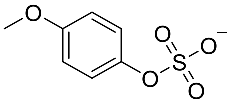		203.0017		1.48	7.09	1	0.000009
6	3-methoxyphenol sulfate	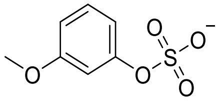	$C_7H_7O_5S^-$	203.0020	203.0014	2.30	9.63	1	0.000138
7	-	-		203.0020		2.96	8.70	2b	0.000126
8	-	-		203.0022		3.94	6.80	3	0.000003
9	-	-	$C_6H_7NO_5S$	203.9976	203.9972	1.96	7.97	3	<0.000001
10							6.24		
11	*Pyrogallol sulfate	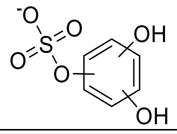	$C_6H_5O_6S^-$	204.9813	204.9812	-0.52	6.83	2b	0.000007
12							7.43		
13	Indoxyl sulfate	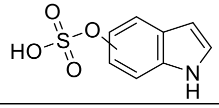	$C_8H_7NO_4S^-$	212.0024	212.0023	-0.55	8.49	2a	0.004877

14	4-(1-propen-1-yl)-, 1-(hydrogen sulfate) phenol	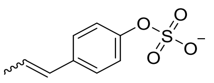	$C_9H_9O_4S^-$	213.0227	213.0222	2.35	7.46	2b	<0.000001
15	Vinylbenzene diol sulfate	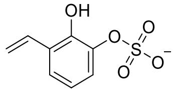	$C_8H_7O_5S^-$	215.0022	215.0020	0.93	9.08	2b	0.023539
16	-	-		215.0023		1.40	10.85	3	<0.000001
17	Tyramine-*O*-sulfate	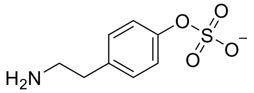	$C_8H_{10}NO_4S^-$	216.0339	216.0331	3.69	5.82	2b	0.018238
18	[4-(2-hydroxyethyl)phenyl]oxidanesulfonic acid	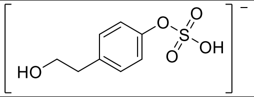	$C_6H_5O_6S^-$	217.0178	217.0171	3.43	11.46	2b	0.000001
19	-	-	-	218.04946	-	-	6.80	3	<0.000001
20	-	-	$C_7H_7O_6S^-$	218.9970	218.9969	0.46	6.38	3	<0.000001
21	-	-					6.78	3	0.000005
22	-	-					7.39	3	0.000585
23	-	-					8.35	3	0.009583
24	-	-	$C_6H_5O_7S^-$	220.9764	220.9761	1.36	6.57	3	0.038557
25							7.12		0.0289
26	-	-	$C_7H_9O_6S^-$	221.0127	221.0125	0.90	8.88	3	0.000001
27	[4-(3-oxopropyl)phenyl]oxidanesulfonic acid	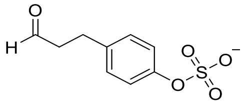	$C_9H_9O_5S^-$	229.0178	229.0171	3.20	11.33	2b	<0.000001
28	(4-butan-2-ylphenyl) sulfate	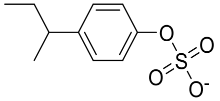	$C_{10}H_{13}O_4S^-$	229.0542	229.0535	-3.16	14.34	2b	0.001806
29	-	-	$C_8H_8NO_5S^-$	230.0131	230.0129	0.87	9.57	3	0.028986 ↓
30	-	-	$C_8H_7O_6S^-$	230.9972	230.9964	3.46	7.00	3	<0.000001
31	Vanillin sulfate	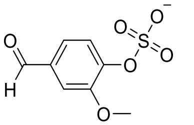		230.9971		-3.27	8.94	1	0.003488
32			$C_7H_5O_7S^-$	232.9764	232.9761	1.29	7.49	2b	<0.000001

33	2-hydroxy-5-(sulfooxy)-benzoic acid	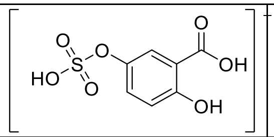					7.22		<0.000001
34	-	-	$C_8H_5O_7S^-$	233.0127	233.0125	0.86	7.20	3	0.00001
35							9.25		0.018029
36	-	-	$C_9H_8NO_5S^-$	242.0130	242.0129	0.41	7.98	3	0.034086 ↓
37	trans-4-Hydroxycinnamic acid sulfate	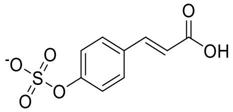	$C_9H_7O_6S^-$	242.9964	242.9964	0.00	9.13	1	0.006048
38	-	-	$C_9H_7O_6S^-$	242.9971	242.9969	0.82	9.42	3	0.031976
39							9.73		
40	-	-	$C_{10}H_{11}O_5S^-$	243.0334	243.0333	0.41	12.48	3	0.000612
41	-	-	$C_8H_6NO_6S^-$	243.9923	243.9921	0.82	6.22	3	0.000016
42				243.9924		1.23	7.03		0.025376↓
43	-	-	$C_{10}H_{13}O_5S^-$	245.0492	245.0489	1.22	11.05	3	0.000279
44	3-methoxy-4-sulfooxybenzoic acid	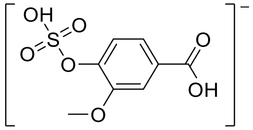	$C_8H_7O_7S^-$	246.9919	246.9913	2.40	7.77	2b	0.000193
45				246.9919		2.56	8.02		<0.000001
46	2-[4-hydroxy-3-(sulfooxy)phenyl]acetic acid	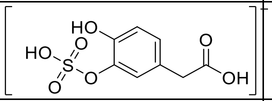	$C_8H_7O_7S^-$	246.9919	246.9913	2.69	6.99	2b	<0.000001
47	-	-	$C_9H_{11}O_6S^-$	247.0283	247.0282	0.40	8.78	3	<0.000001
48	-	-	$C_8H_9O_7S^-$	249.0077	249.0074	1.20	6.62	3	<0.000001
49	-	-		249.0077			7.49	3	0.000004
50	-	-		249.0074		0.00	6.96	3	0.007236
51	Caffeic Acid 3-Sulfate	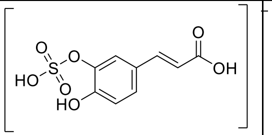	$C_9H_7O_7S^-$	258.9919	258.9913	2.47	9.20	2b	<0.000001
52	-	-	$C_{10}H_{10}O_6S^-$	259.0284	259.0282	0.77	9.45	3	<0.000001
53							12.35		0.00179

54	L-Tyrosine-*O*-sulfate	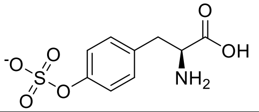	$C_9H_{10}NO_6S^-$	260.0234	260.0229	1.92	5.75	2b	0.037045 ↓
55	Dihydrocaffeic acid sulfate	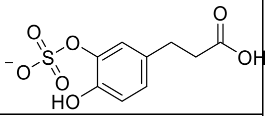	$C_9H_9O_7S^-$	261.0076	261.0069	1.92	2.68	2b	0.034173
56	Homovanillic acid sulfate	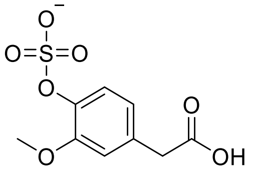		261.0074		-2.01	1.92	1	0.000002
57	-	-	-	261.0440	-	-	9.95	3	<0.000001
58	2-(3,5-dihydroxy-4-sulfooxyphenyl)acetic acid	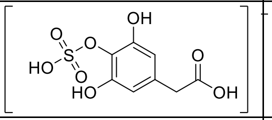	$C_8H_7O_8S^-$	262.9869	262.9862	2.66	7.83	2b	<0.000001
59	-	-	$C_9H_7O_6S^-$	263.0047	-	-	8.73	3	0.000013
60	-	-	$C_6H_9O_{10}S^-$	272.9930	272.9922	2.93	2.62	3	<0.000001
61	Ferulic acid 4-*O*-sulfate	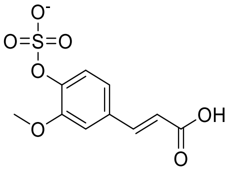	$C_{10}H_9O_7S^-$	273.0075	273.0069	2.20	9.28	1	<0.000001
62	3-[3-Methoxy-4-(sulfooxy)phenyl]propanoic acid	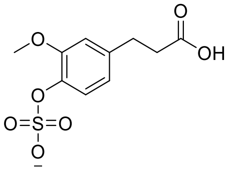	$C_{10}H_{11}O_7S^-$	275.0233	275.0226	2.55	9.47	2b	0.023231
63	-	-	$C_{10}H_{14}NO_6S^-$	276.0550	276.0547	1.09	7.13	3	<0.000001
64	3,5-dimethoxy-4-(sulfooxy)benzoic acid	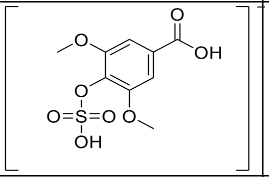	$C_9H_9O_8S^-$	277.0026	277.0018	2.84	7.93	2b	<0.000001
65	-	-	$C_{11}H_{11}O_7S^-$	287.0230	287.0231	-0.35	9.04	3	<0.000001

66	Sinapic acid 4-O-sulfate	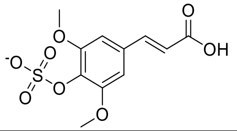	$C_{11}H_{11}O_8S^-$	303.0182	303.0175	2.31	9.12	1	<0.000001
67	-	-	-	303.0723	-	-	7.88	3	0.002569
68				303.0724			7.48		<0.000001
69	-	-	$C_{11}H_{13}O_8S^-$	305.0336	305.0337	-0.33	8.67	3	<0.000001
70				305.0339		0.66	9.00		<0.000001
71	-	-	-	305.0475	-	-	6.20	3	<0.000001
72	Urolithin A sulfate	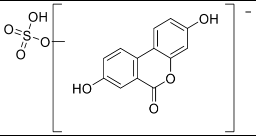	$C_{13}H_7O_7S^-$	306.9921	306.9913	-2.65	12.53	2a	<0.000001
73	-	-	-	309.0554	-	-	8.97	3	0.009094 ↓
74	-	-	$C_{10}H_{13}N_2O_7S^-$	319.0673	-	-	5.95	3	<0.000001
75	-	-	$C_{12}H_{12}NO_8S^-$	330.0291	330.0289	0.61	8.70	3	<0.000001
76	-	-	-	341.1244	-	-	13.45	3	0.001356
77	-	-	-	343.0672	-	-	7.92	3	<0.000001
78	-	-	$C_{16}H_{15}O_7S^-$	351.0571	-	-	2.67	3	<0.000001
79	-	-	$C_{16}H_{22}NO_6S^-$	356.1174	356.1173	0.28	13.20	3	0.016424 ↓
80	-	-	-	373.0463	-	-	7.29	3	<0.000001
81	Enterolactone sulfate	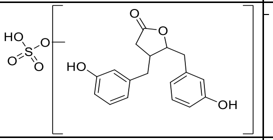	$C_{18}H_{17}O_7S^-$	377.0702	377.0695	-1.80	12.21	2a	<0.000001
82	-	-	$C_{18}H_{21}O_7S^-$	381.1015	381.1013	0.52	11.85	3	<0.000001
83	-	-	-	383.0445	-	-	9.80	3	<0.000001
84	-	-	-	397.1142	-	-	9.08	3	<0.000001
85	-	-	-	421.0600	-	-	13.28	3	0.022027
86	-	-	-	425.0121	-	-	8.47	3	0.010067 ↓
87	-	-	$C_{24}H_{33}O_5S^-$	433.2081	-	-	13.82	3	<0.000001
88	-	-	-	441.1227	-	-	10.87	3	<0.000001
89	-	-	-	447.0935	-	-	11.40	3	0.013738

90				447.0936			10.57		0.008384
91	-	-	-	517.1353	-	-	12.63	3	0.018555

Table S6. All significantly altered glucuronidated metabolites with annotated confidence levels.

(Level 1: Validation with authentic synthetic or commercial standards; Level 2a: Metabolite structure validation based on unambiguous matching of MS^2 spectra with experimental spectra from literature or library sources; Level 2b: Identification of the molecular formula and MS^2 fragmentation pattern comparison using computational tools; Level 3: MS^2-validation of glucuronic acid moiety in the metabolite). *the sum of the peaks has been used for investigation of candidate biomarkers. ↓ marks downregulation at V2 compared to V1.

#	Name	Structure	Chemical formula	m/z Experimental	m/z Theoretical	ppm difference	RT /min	Level of confidence	*P*-value
1	-	-	$C_{12}H_{13}O_8^-$	285.0618	285.0616	0.70	8.60	3	0.000008
2	-	-	-	287.0774	-	-	7.32	3	0.00018
3	4-Ethylphenyl glucuronide	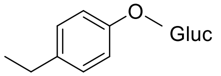	$C_{14}H_{17}O_7^-$	297.0984	297.0975	3.03	12.88	2b	0.039901
4									<0.000001
5	Pyrogallol-*O*-glucuronide	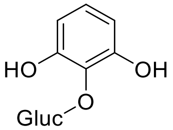	$C_{12}H_{13}O_9^-$	301.0567	301.0560	-2.33	7.22	2b	0.000223
6							8.30		0.007635
7	-	-	$C_{27}H_{27}O_{16}^-$	303.0723	-	-	7.48	3	0.002569
8				303.0724			7.88		<0.000001
9	-	-	-	306.1196	-	-	8.26	3	<0.000001
10	Vitamin B17 (Laetrile)	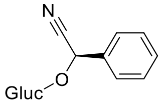	$C_{14}H_{14}O_7^-$	308.0779	308.07705	2.60	7.49	2b	0.000091 ↓
11	-	-	-	317.0517	-	-	6.01	3	0.000002
12	-	-	$C_{14}H_{17}O_7^-$	319.0672	-	-	5.95	3	<0.000001
13				319.0673			6.20		<0.000001
14							6.38		<0.000001
15	-	-	$C_{14}H_{23}O_8^-$	319.1400	319.1398	0.63	13.33	3	0.002106
16				319.1401		0.94	13.68		<0.000001
17	-	-	$C_{14}H_{14}NO_8^-$	324.0728	324.0725	0.93	7.27	3	0.009064 ↓
18	-	-	-	325.1295	325.1293	0.62	13.50	3	0.001243

19	*N*-(2-Hydroxyphenyl)acetamide glucuronide	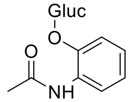	$C_{13}H_{14}NO_9^-$	328.0677	328.0669	2.34	6.92	2b	0.000013
20	-	-	-	329.1608	-	-	14.28	3	<0.000001
21	-	-	$C_{14}H_{22}O_9^-$	335.1350	-	-	11.77	3	0.023538
22	-	-	$C_{15}H_{16}NO_8^-$	338.0884	338.0881	0.89	7.27	3	0.001753 ↓
23	*Eugenol glucuronide	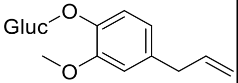	$C_{16}H_{19}O_8^-$	339.1088	339.1080	2.36	12.15	2b	0.001216
24							12.92		0.005634
25	-	-	$C_{14}H_{14}NO_9^-$	340.0677	340.0674	0.88	5.68	3	0.003007
26	Rhododendrol glucuronide	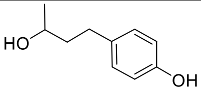 4-(3-hydroxybutyl)phenol	$C_{16}H_{21}O_8^-$	341.1244	-	-	13.07	2b	0.00068
27				341.1244	-	-	13.46	2b	0.001356
28	-	-	$C_{14}H_{16}O_{10}^-$	343.0672	343.0671	-	7.92	3	<0.000001
29	-	-		343.0673		-	7.50	3	<0.000001
30	-	-	$C_{16}H_{22}O_8^-$	343.1400	-	-	13.47	3	0.00075
31	-	-	$C_{16}H_{24}O_8^-$	345.1556	-	-	10.83	3	0.00828
32				345.1557			12.48		0.002069
33							14.25		0.005536
34	-	-	$C_{14}H_{20}O_{10}^-$	349.1142	-	-	9.78	3	<0.000001
35	2-(4-methoxyphenyl)propanoate glucuronide	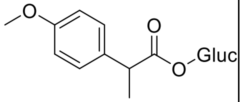	$C_{16}H_{19}O_9^-$	355.1038	355.1035	0.85	9.62	2b	0.001056
36	-	-	$C_{15}H_{19}O_{10}^-$	359.0987	359.0984	0.84	5.83	3	0.024213
37	-	-	$C_{16}H_{22}O_9^-$	359.1350	-	-	11.05	3	0.000002
38	-	-	$C_{16}H_{24}O_9^-$	361.1506	-	-	11.85	3	0.001899
39							12.12		<0.000001
40							12.52		0.000793
41	Ferulic acid glucuronide	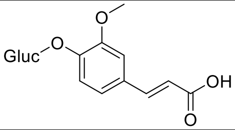	$C_{16}H_{17}O_{10}^-$	369.0830	369.0822	2.05	9.65	2b	<0.000001
42				369.0830		2.09	8.72		<0.000001
43	-	-	$C_{17}H_{21}O_9^-$	369.1194	369.1191	0.81	11.33	3	0.023172

44	Dihydroisoferulic acid 3-glucuronide	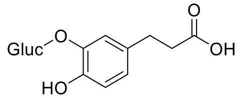	$C_{15}H_{17}O_{10}^{-}$	371.0986	371.0978	2.05	9.13	2b	<0.000001
45				371.0986		2.05	9.39		<0.000001
46	5-(3’,4’-Dihydroxyphenyl)-γ-Valerolactone-4’-O-Glucuronide	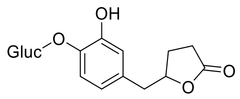	$C_{17}H_{19}O_{10}^{-}$	383.0985	383.0978	1.83	9.06	2b	<0.000001
47							8.75		<0.000001
48	-	-	-	383.1194	-	-	5.88	3	<0.000001
49				383.1197			6.55		<0.000001
50	-	-	$C_{16}H_{23}N_3O_8^{-}$	385.1507	-	-	12.08	3	0.018705
51	Urolithin B glucuronide	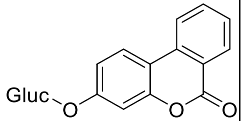	$C_{19}H_{15}O_9^{-}$	387.0723	387.0716	1.87	11.74	3	0.00034
52	-	-	$C_{18}H_{27}O_9^{-}$	387.1663	-	-	12.87	3	0.000007
53	-	-	-	389.1456	-	-	12.08	3	0.000415
54	-	-	-	394.1357	-	-	7.22	3	0.000203 ↓
55	-	-	$C_{18}H_{21}O_{10}^{-}$	397.1142	397.1140	0.50	9.08	3	<0.000001
56	-	-	-	401.1818	-	-	9.75	3	<0.000001
57	*Urolithin A-*O*-glucuronide	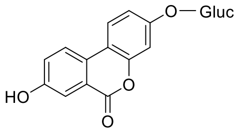	$C_{19}H_{17}O_{10}^{-}$	403.0673	403.0665	1.98	10.41	3	<0.000001
58				403.0673			10.66		0.000008
59	-	-	$C_{18}H_{29}O_{10}^{-}$	405.1767	-	-	12.72	3	<0.000001
60	-	-	-	415.1976	-	-	13.82	3	0.045391
61							14.10		<0.000001
62	Equol 4`-glucuronide	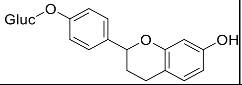	$C_{21}H_{21}O_9^{-}$	417.1193	417.1186	1.79	11.34	3	0.000345
63	-	-	-	419.2289	-	-	15.40	3	0.017618
64	6-[4-(1-carboxyethyl)-3-hydroxy-2-(3-methylbut-2-enyl)phenoxy]-3,4,5-trihydroxyoxane-2-carboxylic acid	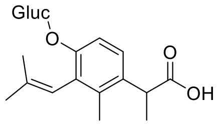	$C_{20}H_{25}O_{10}^{-}$	425.1456	425.1453	0.71	11.73	2b	0.000049
65	-	-	$C_{21}H_{29}O_9^{-}$	425.1819	425.1817	0.47	12.35	3	0.021767

66				425.1820		0.71	13.43		0.00764
67	*Daidzein -*O*-glucuronide	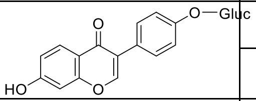	$C_{21}H_{17}O_{10}^{-}$	429.0830	429.0822	1.86	10.90	2b	<0.000001
68			$C_{21}H_{17}O_{10}^{-}$				10.06		
69	-	-	-	429.1769	-	-	13.90	3	0.029096
70							14.40		0.00269
71	-	-	-	431.2286	-	-	13.28	3	0.000025
72	6-[3,5-Dihydroxy-2-(3-phenylpropanoyl)phenoxy]-3,4,5-trihydroxyoxane-2-carboxylic acid	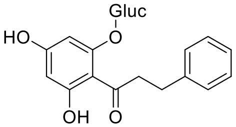	$C_{21}H_{21}O_{10}^{-}$	433.1142	433.1135	1.69	12.16	2b	0.000016
73	-	-	$C_{20}H_{32}O_{10}^{-}$	433.2081	-	-	13.48	3	<0.000001
74							13.82		<0.000001
75	-	-	-	435.2238	-	-	14.40	3	<0.000001
76	-	-	$C_{21}H_{27}O_{10}^{-}$	439.1611	439.1610	0.22	12.42	3	<0.000001
77				439.1612		0.46	10.95		0.008657
78	-	-	-	443.1559	-	-	14.73	3	0.033597
79	-	-	-	452.1927	-	-	12.35	3	0.007978 ↓
80							12.63		0.011717 ↓
81	-	-	-	461.2394	-	-	14.67	3	<0.000001
82	Testosterone glucuronide	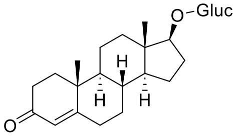	$C_{25}H_{35}O_{8}^{-}$	463.2341	463.2332	1.90	14.84	2b	0.021961 ↓
83	Dihydrotestosterone glucuronide	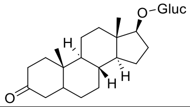	$C_{25}H_{37}O_{8}^{-}$	465.2493	465.2489	0.93	15.15	2a	0.000163 ↓
84	Androsterone glucuronide	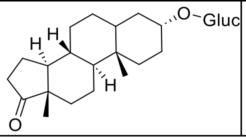	$C_{25}H_{37}O_{8}^{-}$	465.2496	465.2489	1.53	15.66	2b	0.001156 ↓

85	Enterolactone glucuronide	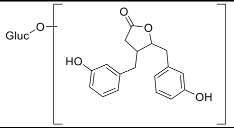	$C_{24}H_{25}O_{10}^-$	473.1452	473.1448	0.92	11.64	2	<0.000001
86	-	-	$C_{24}H_{29}O_{10}^-$	477.1766	477.1766	0.00	11.35	3	<0.000001
87	-	-	$C_{25}H_{38}O_{9}^-$	481.2442	481.2443	-0.21	13.68	3	0.000104 ↓
88	-	-	$C_{24}H_{41}O_{10}^-$	489.2707	489.2705	0.41	15.57	3	<0.000001
89	-	-	$C_{25}H_{35}O_{10}^-$	495.2238	495.2236	0.40	11.25	3	0.00074
90	-	-	$C_{24}H_{40}O_{11}^-$	505.2656	-	-	14.10	3	<0.000001
91	-	-	$C_{27}H_{39}O_{11}^-$	539.2498	539.2498		13.98	3	0.021273 ↓
92							13.50		0.00006 ↓
93	Cortolone-3-glucuronide	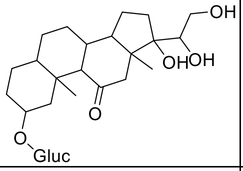	$C_{27}H_{41}O_{11}^-$	541.2654	541.2649	0.88	13.32	2b	0.000033 ↓
94	-	-	-	640.3340	640.3339	0.16	13.12	3	0.010161

Table S7. Input glucuronidated and sulfated metabolites for the ROC analysis.

#	Name
1	p-Cresol glucuronide
2	Daidzein glucuronide
3	Dihydroisoferulic acid glucuronide
4	4-Ethylphenyl glucuronide
5	Enterolactone glucuronide
6	Eugenol glucuronide
7	Isoferulic acid-O-glucuronide
8	Pyrogallol-O-glucuronide
9	Urolithin A glucuronide
10	Urolithin B glucuronide
11	Caffeic acid sulfate
12	Dihydrocaffeic acid sulfate
13	4-Ethylphenyl sulfate
14	Enterolactone sulfate
15	Ferulic acid 4-O-sulfate
16	Homovanillic acid sulfate
17	4-Hydroxycinnamic acid sulfate
18	3-Methoxyphenol sulfate
19	4-Methoxyphenol sulfate
20	Pyrogallol sulfate
21	Resorcinol sulfate
22	Sinapic acid sulfate
23	L-Tyrosine-O-sulfate
24	Tyramine-O-sulfate
25	Urolithin A sulfate
26	Vanillin sulfate

General

All reagents and solvents were purchased from Sigma-Aldrich or Fischer Scientific and were used without further purification. HPLC grade solvents were used for HPLC purification and mass spectrometry grade for UHPLC-ESI-MS analysis. All biochemical reactions were performed with HPLC or LC-MS grade solvents. Solutions were concentrated in vacuo on a Speedvac Concentrator Plus System (Eppendorf, Hamburg, Germany). High-resolution mass spectra were acquired on a Maxis II ETD Q-TOF mass spectrometer (Bruker Daltonics, Germany) using an electrospray ionization (ESI) source with an Elute UHPLC (Bruker Daltonics, Germany) or 1260 Infinity II Binary Pump (Agilent Technologies, USA) system and equipped with a Waters ACQUITY UPLC HSS T3 column (1.8 μm, 2.1 x 100 mm).

Description of procedures

Determination of glucuronidase activity

Glucuronidase activity was tested according to the protocol described by Sigma Aldrich (S9626). In order to calculate the activity of glucuronidase in solution, 65 μL of H_2O were mixed with 50 μL of 75 mM potassium phosphate buffer with 1% (w/v) bovine serum albumin, pH 6.8, 25 μL of 3 mM of phenolphthalein-glucuronide and 10 μL of enzyme test solution. A negative control was also tested, in which no enzyme was added. To stop the reaction, 500 μL of 200 mM glycine buffer, pH 10.4 were added. The resulting solution was transferred to a 96-well plate and the absorbance at 540 nM was measured to monitor the production of phenolphthalein.

At the same time, a phenolphthalein standard curved was prepared, with a ranging quantity of 1-5 μg. The amount of phenolphthalein was plotted against the A540 value and test results were based on the measured absorbance.

The units in solution were calculated using the following equation:

$$Units/mL = \frac{(\text{μg of phenolphthalein released}) \times \text{df}}{\text{V}_\text{E}\ x\ t}$$

Details:

t – Time factor correction (Unit definition for 1 hour)

df – Protein dilution factor

V_E – Volume (in mL) of purified glucuronidase used

Determination of arylsulfatase activity

The arylsulfatase activity assay was based on the assay described for the Helix pomatia arylsulfatase (S9626, Sigma-Aldrich). Briefly, for each enzymatic assay, 65 µL of 200 mM sodium acetate buffer pH 5 and 40 µL of a 6.25 mM aqueous solution of 4-nitrocatechol sulfate were mixed. To this mixture were added 5, 7 or 10 µL of 50 times diluted purified arylsulfatase. At the same time, an assay was performed without any enzyme, as a negative control. The mixtures were incubated for 30 min at 37 °C. After incubation, 500 µL of 1 M NaOH were added to the reaction and the resulting solutions were transferred into a 96-well plate. Their absorbance was measured at a wavelength of 515 nm.

The units in solution were calculated using the following equation:

$$Units/mL = \frac{(\mathrm{A_{Test}} - \mathrm{A_{Blank}}) \times \mathrm{df} \times \mathrm{V_T}}{\varepsilon_{515}\ x\ \mathrm{V_E}\ x\ t}$$

Details:

A_{Test} – Absorbance measured for the test solutions at 515 nm

A_{Blank} – Absorbance measured for the blank at 515 nm

t – Time factor correction (Unit definition for 1 hour)

df – Protein dilution factor

V_T – Total volume (in mL) of the assay

ε_{515} – Milimolar extinction coefficient of p-nitrocatechol at 515 nm ($\mu m^{-1}cm^{-1}$)

V_E – Volume (in mL) of purified arylsulfatase used
